# Inorganic nanoparticle-based treatment approaches for colorectal cancer: recent advancements and challenges

**DOI:** 10.1186/s12951-024-02701-3

**Published:** 2024-07-19

**Authors:** Soumya Narayana, B.H. Jaswanth Gowda, Umme Hani, Sharmin Sultana Shimu, Karthika Paul, Avinaba Das, Sumel Ashique, Mohammed Gulzar Ahmed, Maryam Abbasi Tarighat, Gholamreza Abdi

**Affiliations:** 1 Department of Pharmaceutics, Yenepoya Pharmacy College & Research Centre, Yenepoya (Deemed to be University), Mangalore, 575018 Karnataka India; 2 Department of Pharmaceutics, College of Pharmacy, King Khalid University, Abha, 61421 Saudi Arabia; 3 Department of Genetic Engineering and Biotechnology, University of Rajshahi, Rajshahi, 6205 Bangladesh; 4 Department of Pharmaceutical Chemistry, JSS College of Pharmacy, JSS Academy of Higher Education and Research (JSSAHER), Mysuru, 570015 Karnataka India; 5 Department of Pharmaceutical Sciences, Bengal College of Pharmaceutical Sciences & Research, Durgapur, 713212 West Bengal India; 6 Faculty of Nano and Bio Science and Technology, Persian Gulf University, Bushehr, 75169 Iran; 7 Department of Biotechnology, Persian Gulf Research Institute, Persian Gulf University, Bushehr, 75169 Iran; 8 School of Pharmaceutical Sciences , Lovely Professional University, Phagwara, Punjab 144411, India

**Keywords:** Colon cancer, Nanotechnology, Nanoparticles, Inorganic nanoparticles, Drug delivery, Photothermal therapy, Photodynamic therapy

## Abstract

Colorectal cancer, the third most prevalent cancer globally, contributes significantly to mortality rates, with over 1.9 million reported cases and nearly 935,000 fatalities annually. Surgical resection is a primary approach for localized colorectal tumors, with adjunct therapies like chemotherapy, radiotherapy, and targeted/immunotherapy considered depending on the tumor stage. However, despite preferences for targeted and immunotherapy post-surgery, chemotherapy remains commonly chosen due to its lower cost and high cancer-killing efficiency. Yet, chemotherapy faces issues such as tumor resistance and severe side effects. Nanotechnology has emerged in cancer therapy by alleviating the drawbacks of current treatment approaches. In the past few decades, inorganic nanoparticles have shown promise in combating colorectal cancer, offering advantages over conventional chemotherapy. Compared to organic nanoparticles, inorganic nanoparticles exhibit properties like photosensitivity, conductivity, magnetic allure, and thermal proficiency, allowing them to function as both drug carriers and therapeutic agents. Derived primarily from carbon, silica, metals, and metal oxides, they offer superior drug-loading capacity, heightened quantum yield, and participation in advanced photothermal and photodynamic therapies. This review provides a brief overview of the pathophysiology of colorectal cancer and the pivotal role of inorganic nanoparticles in photothermal therapy photodynamic therapy, and drug delivery. Additionally, it discusses numerous inorganic nanoparticles in colorectal cancer therapy based on recent literature.

**Graphical abstract:**

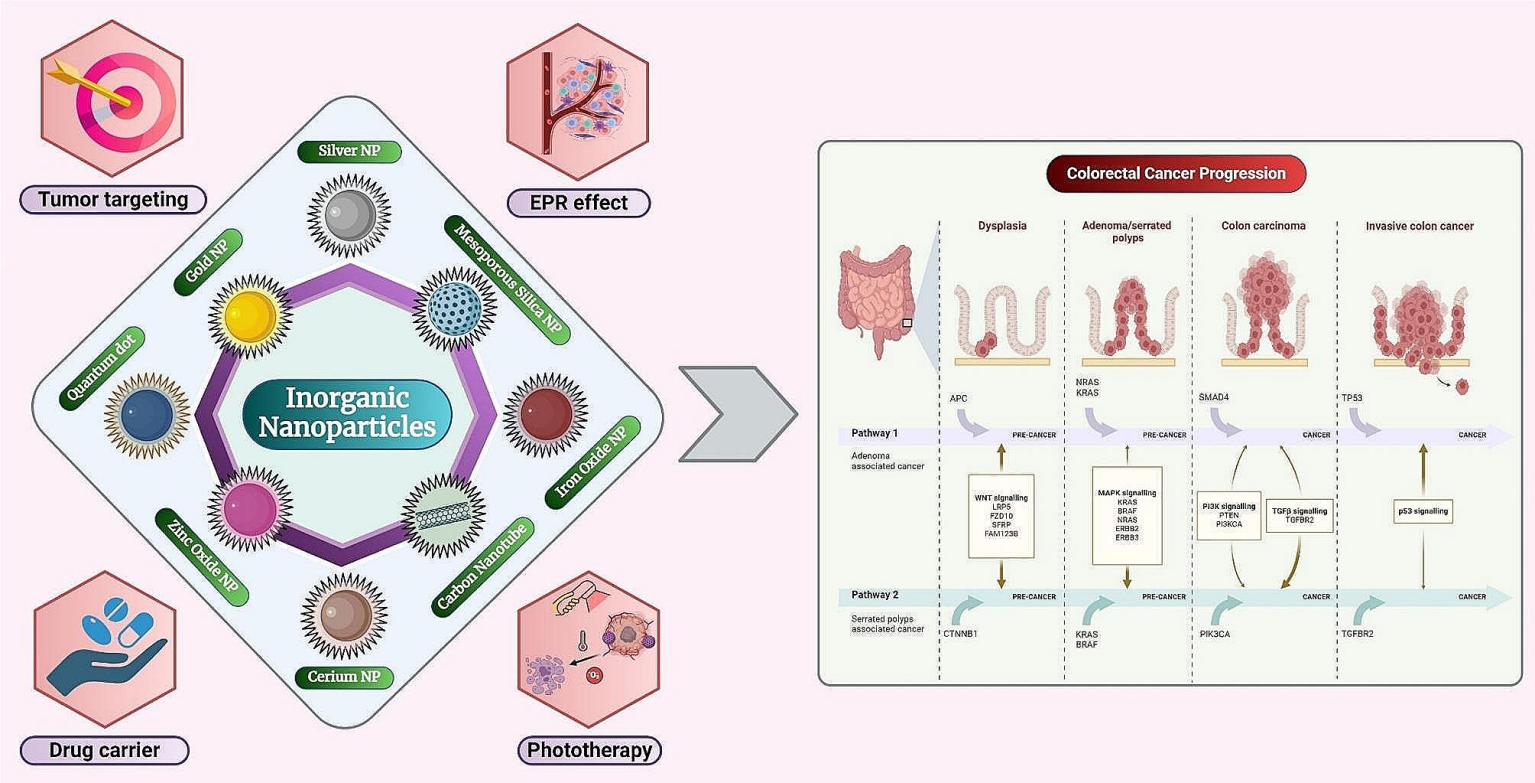

## Introduction

Colorectal cancer (CRC) ranks as the third most prevalent form of cancer worldwide, representing a significant contributor to mortality rates [1]. Data from Globcan (2020) reveals a staggering 1,931,50 reported cases of CRC, with 935,173 resulting in fatalities across all genders and age groups on a global scale [1, 2]. The colon and rectum, integral components of the digestive system, constitute the terminal segment of the intestinal tract. Stretching approximately one meter, their journey commences at the ileocecal valve, marking the conclusion of the small intestine, and culminates at the anus [3–5]. Colon cancer targets the epithelial lining of the colon, a tissue typically replenished every five days through the division of colonic epithelial stem cells. CRC emerges as the culmination of a gradual succession of progressive stages in colon neoplasia, evolving over an extended duration [6].

The approach to treating colorectal cancer is heavily influenced by the cancer’s stage, ranging from I to IV. For early-stage cancer (Stage I), surgical options such as polypectomy, endoscopic mucosal resection, or minimally invasive laparoscopic surgery are commonly used. When the cancer progresses to Stage II, infiltrating the colon wall without affecting the lymph nodes, a partial colectomy is typically performed, frequently using laparoscopic techniques, along with the removal of nearby lymph nodes. In Stage III, where cancer has spread to lymph nodes, treatment generally involves a combination of partial colectomy and chemotherapy to destroy any remaining cancer cells and lower the chances of recurrence [7]. For Stage IV cancer, which involves metastasis to distant organs like the liver or lungs, a more intensive treatment regimen is necessary, including surgery, chemotherapy, targeted therapy, and immunotherapy to control and manage the disease. Additionally, radiation therapy is employed to reduce tumor size and relieve symptoms when surgery is not an option [8, 9]. Despite the preference for immunotherapy and targeted therapy post-surgery, their limitations such as limited bioavailability and high costs often steer patients towards chemotherapy [10]. Although chemotherapy presents a notable reduction in treatment expenses, its efficacy is significantly compromised by factors such as tumor resistance, poor solubility, and permeability, limited bioavailability, lack of target specificity, and associated severe side effects [11].

**Fig. 1:**
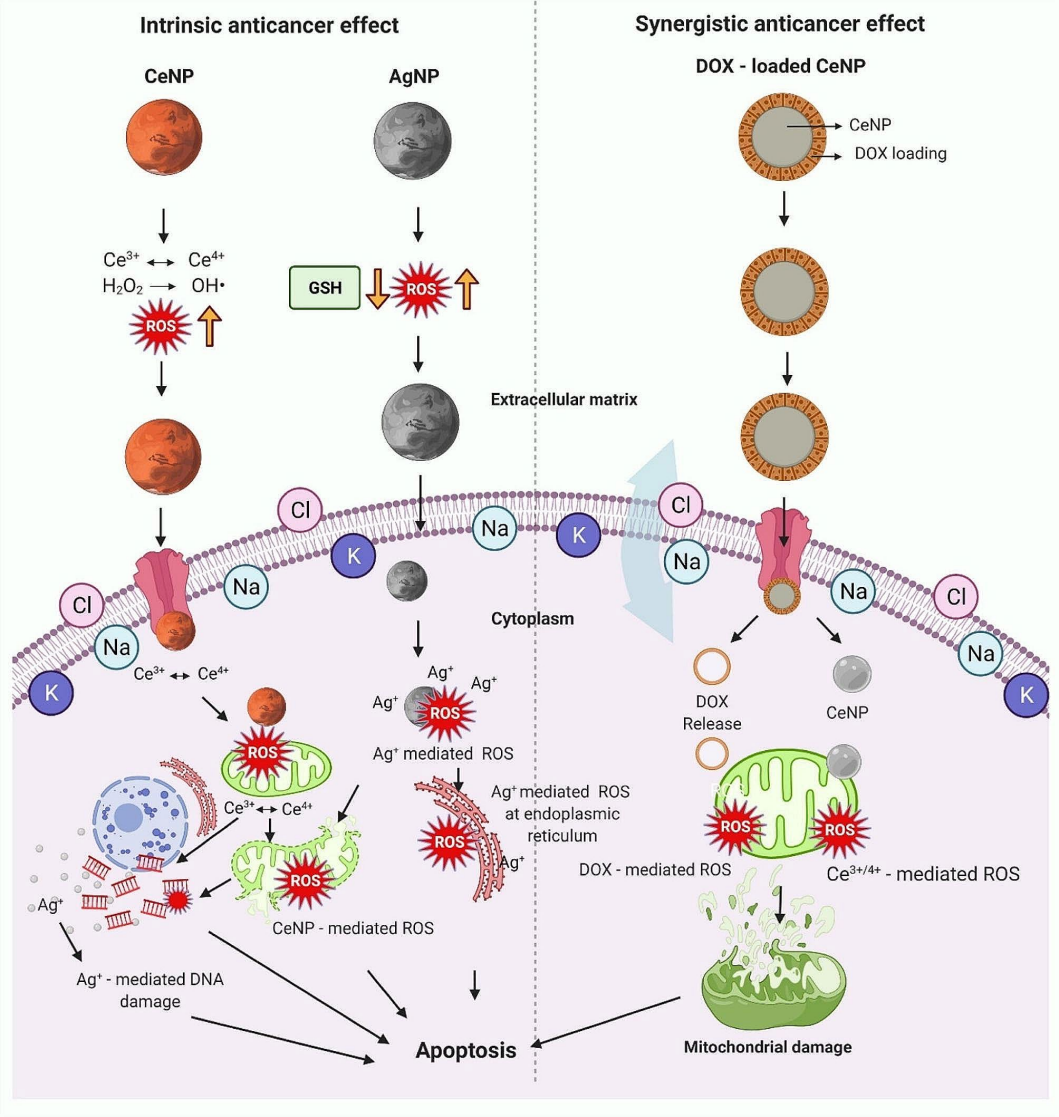
Intrinsic and synergistic anticancer effects of inorganic nanoparticles (INPs) in cancer treatment. Silver nanoparticles (AgNPs) induce apoptosis in cancer cells initially through reactive oxygen species (ROS) production and consequent reduction in the antioxidant glutathione (GSH). Subsequently, the release of Ag + ions increase the ROS level, which damages mitochondria, DNA, and the endoplasmic reticulum. Ceria nanoparticles (CeNPs) induce apoptosis in cancer cells by generating ROS through pH-induced Ce3+ ↔ Ce4 + redox, which damages mitochondria and DNA. When combined with doxorubicin (DOX), CeNPs exhibit a synergistic effect on cancer cells while protecting normal cells owing to their unique prooxidant-antioxidant activity, reproduced with permission from [32], copyright 2022, Elsevier

Nanotechnology, an emerging field at the intersection of science and engineering, focuses on manipulating materials at the nanoscale, typically ranging from 1 to 1000 nm in size [12–17]. Within this realm, several nanomaterials exhibit tremendous potential to enhance the efficacy of cancer therapies by delivering anticancer drugs to the tumor site [18–23]. Nanoparticles (NPs) possess distinct characteristics, including their ability to passively target tumors through mechanisms like the enhanced permeability and retention (EPR) effect, evade the body’s reticuloendothelial system (RES), and facilitate improved permeation through tissues [24–29]. In recent times, the spotlight in cancer therapy research has shifted towards inorganic NPs (INPs), captivating scientists owing to their distinctive physicochemical traits, contingent upon material and size [30, 31]. Unlike organic NPs, INPs boast unparalleled properties such as exceptional photosensitivity, remarkable conductivity, optical prowess, magnetic allure, and thermal proficiency, serving dual roles as both carriers for drugs and therapeutic agents themselves **(**Fig. 1**)** [32]. Their ease of synthesis, large surface area, and mechanical and chemical stability underscore their superiority. Typically derived from metals, metal oxides, and non-metallic materials (carbon and silica), INPs offer a myriad of advantages as carriers for drugs, including heightened quantum yield and drug-loading capacity, as well as the ability to participate in PTT and PDT [33]. INPs offer significant advantages in cancer treatment through photothermal therapy (PTT) and photodynamic therapy (PDT). INPs generate localized heat in PTT and produce reactive oxygen species in PDT, enhancing precision and minimizing damage to healthy tissues. Their higher absorption coefficients, stability, and prolonged circulation improve treatment efficacy. Additionally, INPs serve as effective drug delivery systems upon surface modification/engineering, providing controlled release and multifunctional capabilities for bioimaging and therapy, making them promising candidates for advanced cancer treatments [33].

To date, there are only two INPs approved for cancer therapy. In 2010, the European Medicines Agency (EMA) approved the first INP, iron oxide NPs (Fe_3_O_4_NPs) (marketed as NanoTherm^®^ by MagForce Nanotechnologies AG), for the treatment of glioblastoma, pancreatic, and prostate cancer through thermal ablation using a magnetic field. Subsequently, in 2019, the EMA granted its initial approval for hafnium oxide NPs (marketed as Hensify^®^ [NBTXR3] by Nanobiotix) for radiotherapy in the treatment of locally advanced soft tissue sarcoma [34]. However, there are currently no specific INPs utilized for the treatment of CRC in the market, despite its mortality rate ranking second among all other types of cancer. This suggests that there is still potential for various other INPs to be explored, such as mesoporous silica nanoparticles (MSNs), cerium oxide NPs (CeONPs), silver NPs (AgNPs), zinc oxide NPs (ZnONPs), gold NPs (AuNPs), carbon nanotubes (CNTs), etc., for the effective treatment of CRC [32]. In this context, this review provides brief information on the pathophysiology of CRC, the role of INPs in drug delivery, photothermal therapy (PTT), and photodynamic therapy (PDT). Furthermore, it meticulously discusses the importance of several INPs in CRC therapy based on recent literature.

## Pathophysiology of colorectal cancer

**Fig. 2:**
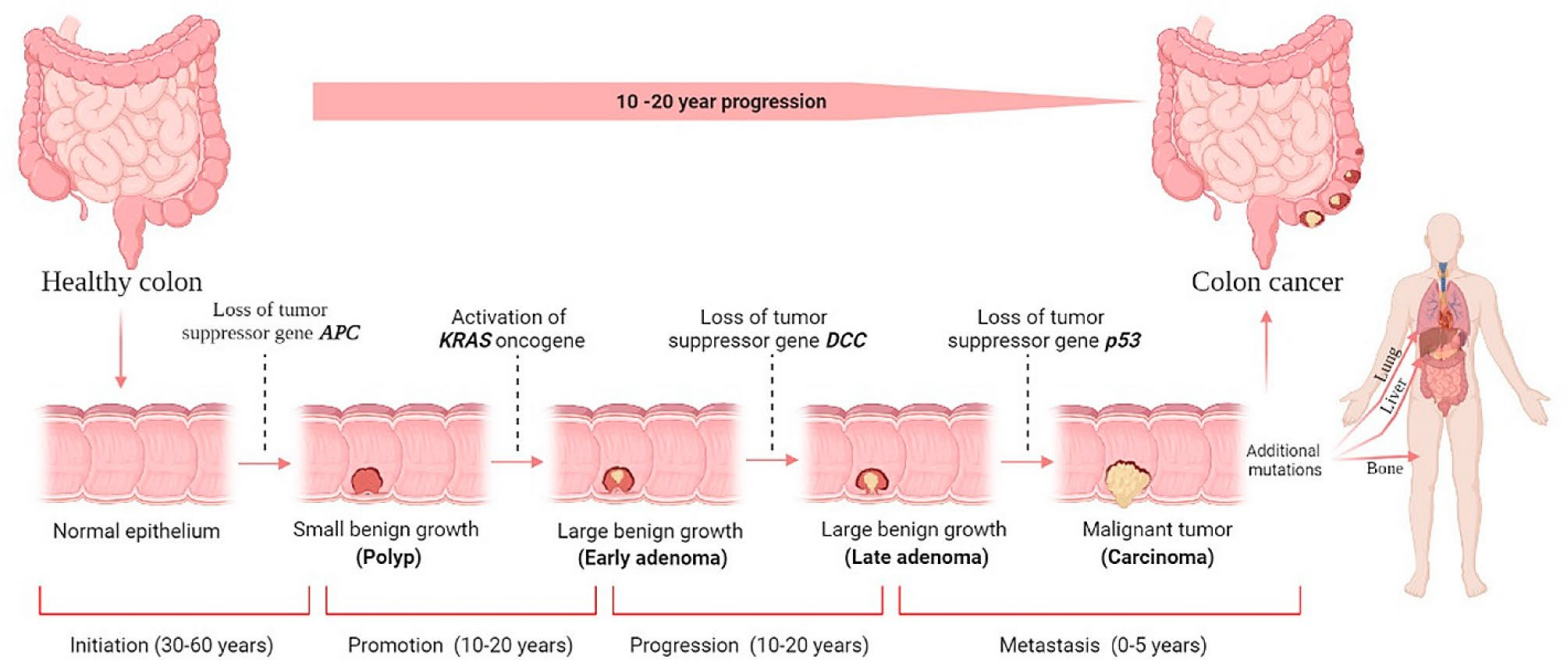
Colorectal cancer stages and development. There are four stages in the development of colorectal cancer carcinogenesis: initiation, promotion, progression, and metastasis. The liver is the most common metastatic site, followed by the lung and bone. Although it is difficult to determine the duration required for each stage, decades will likely be required to form colorectal cancer, reproduced with permission from [35], copyright 2022, MDPI.

The progression from normal colonic epithelium to a precancerous lesion (adenoma), and eventually to invasive carcinoma, necessitates the accumulation of genetic mutations, whether acquired somatically or inherited germline **(**Fig. 2**)** [35]. The theory of colonic carcinogenesis revolves around a clonal evolution of mutations, granting cells a survival advantage and immortality, thereby facilitating the development of additional mutations that contribute to cancer hallmarks such as invasion, proliferation, metastasis, etc. Observational data in clinical settings indicate that CRC often originates from adenomatous polyps, which typically undergo dysplastic alterations for 10 to 20 years before progressing to invasive carcinoma [36]. Early identification and removal of these polyps have been demonstrated to lower the incidence of CRC. CRC entails a complex interplay of molecular pathways, among which three major pathways stand out: chromosomal instability (CIN), microsatellite instability (MSI), and CpG island methylator phenotype (CIMP). These pathways collectively contribute significantly to CRC development through a combination of genetic and epigenetic alterations [37]. These intricate molecular pathways involved in CRC can influence both tumor progression and metastatic potential, with variations observed in epidemiological patterns, mutational profiles, and immune responses depending on the specific pathway engaged. CIN stands out as the predominant feature in the majority of CRC, constituting approximately 80–85% of cases. CIN manifests through the activation of growth-promoting pathways coupled with a concurrent reduction in apoptotic pathway activity, with the latter being particularly prevalent. These tumors typically originate from adenomatous polyps, primarily triggered by mutations that deactivate the adenomatous polyposis coli (APC) gene [38]. Cells deficient in DNA mismatch repair (dMMR), often due to mutations in genes such as MLH1 or MSH2, experience a buildup of genomic errors leading to pronounced microsatellite instability (MSI-H), a characteristic feature of Lynch syndrome. CpG hypermethylation of DNA can either activate or suppress the expression of specific genes, such as BRAF and MLH1, respectively. Additionally, somatic mutations in sporadic oncogenes like RAS, SRC, and MYC have been implicated in CRC, with RAS mutations, particularly, holding significant clinical relevance. These mutations, encompassing variants such as HRAS, KRAS, and NRAS, are detected in approximately 50% of sporadic CRC cases [39]. Currently, they are being leveraged for CRC screening through stool-DNA testing and are associated with the lack of response to epidermal growth factor receptor (EGFR) targeted therapy, thus prompting the exploration of potential direct targeted agents [36].

## Inorganic nanoparticles in cancer therapy

Before delving into the specifics of each type of INP-based cancer therapy, we will first explore the potential of INPs in PTT, PDT, and as carriers for anticancer agents. These discussions will set the stage for a comprehensive understanding of how INPs can significantly enhance cancer treatment approaches [32].

### Photothermal therapy

Hyperthermia-based cancer treatments involve subjecting the targeted tissue to elevated temperatures, capable of inducing cancer cell death (thermal ablation, occurring at temperatures above 45 °C) or enhancing cancer cell sensitivity to other therapeutic interventions (mild hyperthermia, temperatures ranging between 40 and 45 °C) [40]. In PTT, NIR-II wavelengths provide superior tissue penetration and enhanced safety compared to NIR-I (750–900 nm), owing to decreased scattering and absorption by biological tissues. Consequently, NIR-II (1000–1700 nm) is more effective for targeting deeper tumors and reducing harm to adjacent healthy tissue [41]. In conventional hyperthermia approaches, temperature elevation in the target tissue is typically achieved through external means, such as regional hyperthermia, superficial hyperthermia, and whole-body hyperthermia utilizing thermal baths, microwaves, or radiofrequency [42, 43]. However, this often results in a temperature gradient, peaking at the body surface and diminishing with distance from the external heat source. Consequently, healthy tissues may also be subjected to temperature elevation, leading to undesirable side effects [41, 44]. Thus, to overcome these drawbacks, researchers have directed their efforts toward developing more efficient approaches, particularly those capable of inducing localized temperature increases confined to the tumor site.

NPs exhibiting the ability to generate heat in response to external stimuli have emerged as promising candidates, addressing the limitations of conventional hyperthermia methods [33]. Notably, the size of NPs enables them to exploit the abnormal vasculature of tumors, facilitating their accumulation within the tumor tissue via mechanisms such as the EPR effect or vascular bursts [45, 46]. Subsequently, these NPs can facilitate localized thermal destruction of cancer cells upon exposure to external stimuli such as NIR radiation, thereby minimizing damage to surrounding healthy tissues. The use of NIR, particularly in the NIR-I and NIR-II ranges, is vital for NP-driven hyperthermia [42, 47]. This is because biological elements have minimal absorption in this spectrum, leading to fewer off-target interactions and increased penetration within the human body, ultimately amplifying therapeutic effectiveness [48].

Both inorganic and organic NPs have been extensively explored in PTT for cancer treatment. However, INPs offer distinct advantages over their organic counterparts. Notably, INPs tend to possess higher absorption coefficients than organic NPs, facilitating a more efficient conversion of light into heat [49, 50]. Moreover, their inherent stability and prolonged circulation in the body can enhance tumor accumulation and improve treatment efficacy. Additionally, organic NPs generally exhibit lower absorption coefficients compared to INPs, thus constraining their effectiveness in PTT [51].

### Photodynamic therapy

PDT, heralded as an innovative method for tumor ablation, presents a nuanced approach with minimized long-term morbidity. By orchestrating the interplay of three essential elements such as light, molecular oxygen (O_2_), and a photosensitizer. PDT generates cytotoxic reactive oxygen species (ROS) that selectively exterminate cancer cells [40]. While PDT has demonstrated considerable efficacy in treating specific cancers like skin and oral cancers, its widespread adoption as a primary therapeutic option in clinical settings remains challenging due to several inherent limitations. These include the absence of an optimal photosensitizer with superior tumor selectivity and efficacy in generating ROS, as well as the lack of robust methodologies for determining appropriate light dosimetry and monitoring treatment response effectively [52–54]. Notably, the absence of an ideal photosensitizer stands out as the primary obstacle hindering the progress and broader application of PDT. Despite the utilization of various small organic PSs, such as porphyrin structures, synthetic dyes, and natural products in clinical settings, their efficacy against cancers is often limited by inherent drawbacks such as poor water solubility, inadequate photostability, low extinction coefficient, limited absorption in the NIR region, suboptimal generation of singlet oxygen (^1^O_2_), and inadequate cancer selectivity [55].

Recently, INPs made of metals, carbon materials, and silica, offer an innovative avenue to enhance the effectiveness of current photosensitizers and tackle obstacles in cancer PDT [56]. INP-based photosensitizers signify a substantial leap forward compared to traditional organic photosensitizers. Their benefits are multifaceted: firstly, they exhibit substantial extinction coefficients, facilitating the efficient transfer of energy for photosensitization [57]. Secondly, their surfaces are easily adaptable, allowing for the attachment of target ligands and functional groups, thus amplifying their selectivity towards tumor cells. Moreover, their diminutive size grants them expansive surface-to-volume ratios, leveraging the EPR effect to accumulate more effectively within solid tumor tissues [58]. Additionally, they seamlessly integrate with chemo drugs and imaging modalities, enabling the implementation of precision-guided treatment approaches. Remarkably, specific inorganic nanomaterials possess distinctive optical properties, enabling them to act as direct photosensitizers, and catalyzing the production of singlet oxygen (^1^O_2_) upon exposure to light, eliminating the necessity for traditional organic photosensitizers. This capability not only ensures superior ^1^O_2_ quantum yields but also confers robust resistance to photobleaching, all while maintaining significant extinction coefficients [59, 60].

### Drug delivery

In cancer treatment, various organic NPs such as liposomes, micelles, and polymer-based nano-drug delivery systems have progressed to advanced developmental stages, with several even securing FDA approval [61]. Nevertheless, these conventional nanocarriers confront hurdles like drug leakage and uncontrolled release rates. Recent strides in synthesis methodologies have catalyzed the emergence of INP-based drug delivery systems, predominantly in the pre-clinical phase [62, 63]. Yet, their synthesis simplicity and modifiability empower precise regulation of size, morphology, and surface characteristics. INPs present a gamut of opportunities as integrated and versatile platforms for both bioimaging and drug delivery, harnessing their optical, electronic, and magnetic attributes [32]. Given these advantages, INPs like MSNs and CNTs serve as dedicated drug carriers. However, quantum dots (QDs), AuNPs, AgNPs, and others function not only as drug carriers but also as therapeutic agents, capitalizing on their potential in cancer therapy, making them interesting candidates in CRC treatment [64].

## Inorganic nanoparticle-based approaches for colorectal cancer therapy

INPs have become the focal point of recent cancer therapy research. This shift has captivated scientists as they delve into the distinctive physicochemical properties of these particles, which are contingent upon factors such as material composition and size. These are derived from metal oxides (e.g., iron, manganese, zinc, etc.), metals (e.g., gold, silver, etc.), carbons (e.g., carbon dots, CNTs, etc.), semiconductors (QDs), etc. and extensively explored as a therapeutic tool in the treatment of various cancer conditions [65, 66]. The various inorganic NPs include MSNs, QDs, CNTs, AuNPs, Fe_3_O_4_NPs, AgNPs, and ZnONPs [67]. For instance, the QDs have unique features like high fluorescence with a higher emission range, which makes them suitable for bioimaging [68]. Metal oxide NPs exhibit greater catalytic ability for organic reactions by declining the active energy of those reactions [69, 70]. Their key features are biocompatibility, precise targeting, non-toxic, thermostability, unique optical properties, small size, enhanced surface area, controllable structure, magnificent bioavailability, and physicochemical properties [71]. Due to their distinctive properties and surface modification/engineering potential, they have been widely explored as promising candidates in CRC therapy. Additionally, comprehensive descriptions of the latest research work concerning the utilization of INPs in combating CRC are further elaborated upon in the subsequent sections.

### Carbon nanotubes

CNTs are a unique type of NPs which are minute tube-shaped materials that contain carbon atoms in their nanostructure, which looks like a honeycomb lattice. Generally, based on the sheet number of carbon atoms, they are divided into single-walled CNTs (SWCNTs) and multi-walled CNTs (MWCNTs) [72]. Due to their distinctive physicochemical properties, CNTs are considerably designed for cancer therapy. These unique nanomaterials help cancer imaging and drug delivery to tumor sites [73]. Several studies reported that due to the needle-shaped architecture of CNTs, they are quickly taken up by solid tumors [74]. Over recent years CNTs have been utilized to locate the solid tumors of the colon and rectum [75]. One study disclosed that the collaboration of SWCNTs with TRAIL enhanced the cell apoptosis in colorectal cell lines around ten times greater than TRAIL alone [76]. Several properties of CNTs, like high surface area, chemical stability, and thermal and electrical conductivity due to this CNTs could be versatile nanomaterials in the treatment of CRC [77].

In a recent investigation, Gonzalez and colleagues developed functionalized SWCNTs with nanocrystalline cellulose II (II NCC) colloidal system platforms to enhance the activity in colon cancer therapy. The TEM images disclosed that the dispersion of SWCNTs/NCC hybrids resulted in a rod-shaped nanocrystal structure. The SWCNTs/NCC containing combinations resulted in efficient size and width. The nanohybrids do not affect normal cells and considerably decrease the tumor cells. These nanohybrid platforms and Capecitabine potency were analyzed using IC50 value against Caco-2 cells. The outcomes indicated that the IC50 value of 1a-SWCNT (0.201 ± 0.115) and 2a-SWCNT (0.113 ± 0.115) is less than that of Capecitabine (0.279 ± 0.110). The results disclosed that the same anti-cancer effects resulted with a lower dose than the capecitabine drug alone. The authors concluded that NCC stabilized SWCNTs in aqueous dispersion, resulting in nanohybrids showing no toxicity. Aqueous dispersions of fluorescein functionalized SWCNTs/NCC II exhibited enhanced intrinsic activity against Caco 2 cells compared with the non-functionalized chemotherapeutic drug capecitabine [78].

**Fig. 3:**
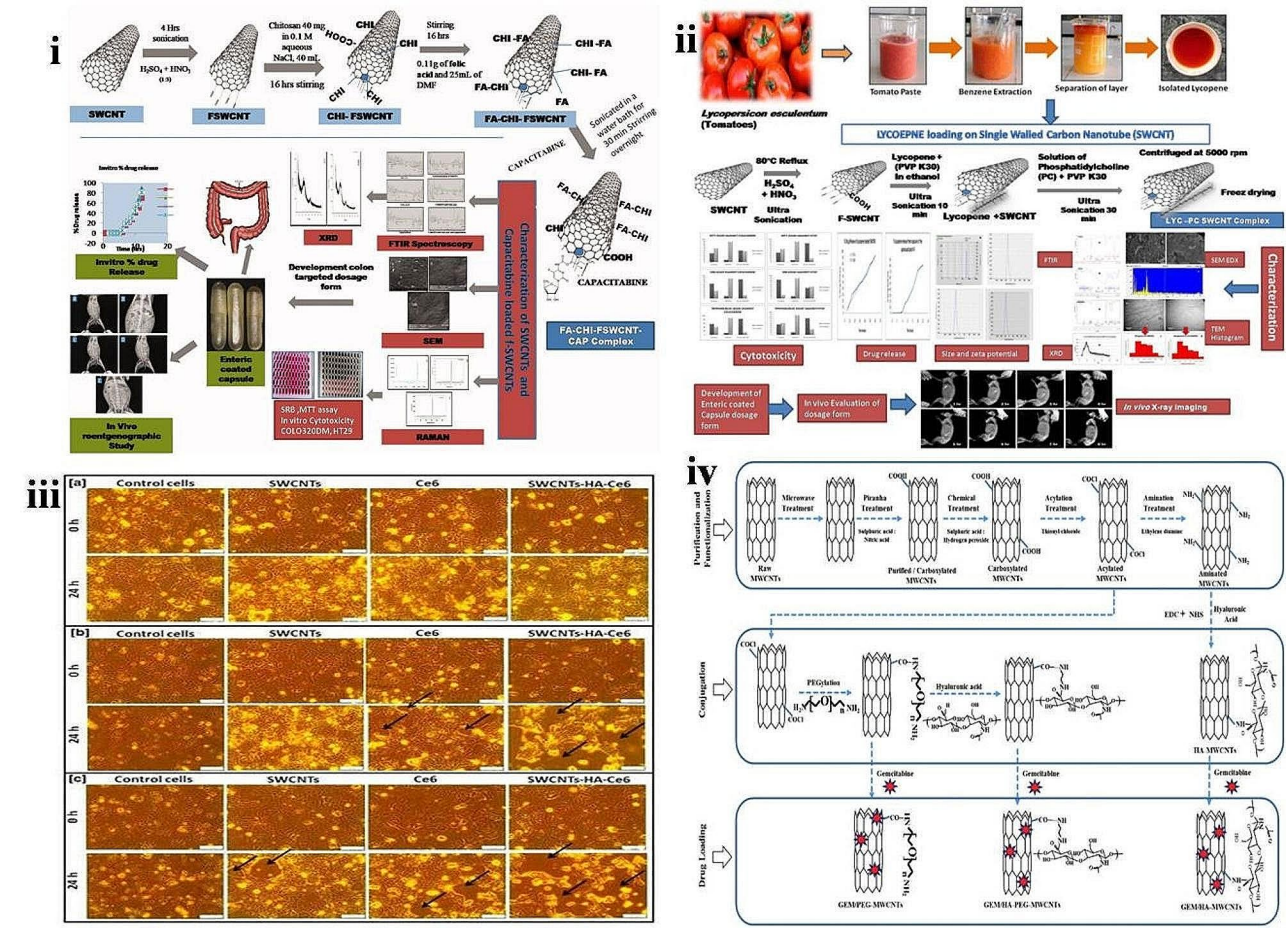
(**i**) Conceptual illustration of the preparation of FA–CHI-F-SWCNT-Capecitabine and characterization reproduced with permission from Ref. [81], (**ii**) Graphical representation of lycopene isolation, loading to SWCNTs and characterization reproduced with permission from Ref. [82], (**iii**) Macroscopic images of Caco 2 cell lines using PDT at a fluence of (**a**) untreated cells at 0 and 24 h, (**b**) 5 J/cm^2^ at 0 and 24 h **c** 10 J/cm^2^ at 0 and 24 h, reproduced with permission from Ref. [84] (**iv**) Schematic representation of purification and drug loading into MWCNTs reproduced with permission from Ref. [86]

Similarly, 5-Fluorouracil (5-FU) is a well-known chemotherapeutic agent against colorectal tumors, but it is associated with several drawbacks [79], and there was a need to develop a prodrug. Capecitabine is a prodrug of 5-FU, and this has been designed to overcome limitations associated with the 5-FU [80]. To check their efficacy, Randive and teammates developed chitosan (CHI) and folic acid (FA) functionalized CNTs (fCNTs) containing Capecitabine against colon cancer cells. The Raman spectroscopy, FTIR, and X-ray diffraction studies confirmed the conjugation of Capecitabine in CNTs. SEM studies showed the size range of CNTs around 200–500 nm, and drug loading capacity was around 94.63 ± 1.07%. The cytotoxicity study using COLO320DM cells showed % inhibition of 86.45 ± 0.5788% for FA–CHI-fSWCNT- Capecitabine, and 50.52 ± 0.3106% for pure drug. Against HT29, the % inhibition was observed to be 82.76 ± 0.4668% (FA–CHI-F-SWCNT-Capecitabine) and 56.41 ± 0.2316% (pure drug). In vivo studies in rabbit models revealed that the developed novel system targets the drug specifically in the colon region without premature release. Hence, the research team concluded that the developed system facilitates drug delivery to colon, rectal, and intestinal cancers **(**Fig. 3**(i))** [81].

SWCNTs emerged as a novel therapeutic tool to optimize the efficacy of many drugs. Despite its excellent properties, this is limited because its poor solubility leads to reduced bioavailability, absorption, blood transportation, and secretion. To overcome the above limitations, Shejawal and co-workers developed functionalized CNTs (fSWCNTs) of isolated lycopene (LYC) for its targeted release in colon cancer cells. The Phosphatidylcholine (PC) and Polyvinylpyrrolidone (PVP) K30 were used as stabilizing agents. SEM studies showed that LYC-PC-PVP-fSWCNTs exhibited a size range of 10–300 nm. TEM studies confirmed normal size distribution in the developed novel system was 272 nm with a ζ–potential of -40mV. The resulting novel approach exhibited 90 ± 0.256% of the loading capacity of LYC and 93.81 ± 1.2172% of %CDR in 60 min. The cytotoxicity activity against COLO320DM and HT 29 was better than pure lycopene. The results of in vitro and in vivo studies revealed that the drug targeted the site of the colon entirely without any premature release **(**Fig. 3**(ii))** [82]. PDT is a well-known cancer therapy, and chlorine e6 (Ce6) is a naturally derived photosensitizer (PS) that is commonly used in PDT. The solubility of PS is the major challenge in PDT [83]. To overcome this issue, Sundaram and Abrahamse synthesized nano biocomposites of hyaluronic acid (HA) coupled and Ce6-coated SWCNTs for PDT to treat colon cancer. The average particle size of nano biocomposite was increased in SWCNTs-HA-Ce6 (203 ± 6.6 nm) compared to plain SWCNTs (191 ± 4.6 nm). The ζ–potential analysis indicated that developed MWCNTs (− 17.8 ± 1.2 mV) and SWCNTs-HA-Ce6 (− 18.9 ± 1 mV) are stable. The SWCNTs-HA-Ce6 exhibited a significantly higher percentage of cell death **(**Fig. 3**(iii))** in irradiated Caco-2 cells (84.9%). The authors concluded that the novel synthesized nano biocomposite (SWCNTs-HA-Ce6 ) intensified the ability of PDT and induced the death of colon cancer cells [84].

Conventional treatment modes have limited therapeutic efficacy for colon cancer due to the lack of specificity and selectivity against colon cancer cells. Hence, site-specific drug delivery to colon tumor cells is needed, which upturns the concentration at the targeted site and minimizes the dose and side effects [85]. To overcome this, Prajapati and colleagues developed Gemcitabine (GEM) MWCNTs (plain), coupled with HA (GEM/HA/MWCNTs), PEGylated (GEM/PEG-MWCNTs) and HA coupled and PEGylated (GEM-HA-PEG-MWCNTs) **(**Fig. 3**(iv))** as a novel delivery to an intracellular system and evaluation its efficacy in vitro and in vivo models. The effectiveness of the current approach was compared with the free GEM. The FT-IR study confirmed the HA and PEG conjugation on MWCNTs. TEM images confirmed tubular shape open-ended structure with nano size for purified MWCNTs and GEM/HA-MWCNTs. The average particle size and PDI of GEM-MWCNTs (28 ± 2.1 nm & 0.412 ± 0.034), GEM/HA-PEG-MWCNTs (39 ± 1.9 nm & 0.192 ± 0.053), GEM/HA-MWCNTs (32 ± 2.4 nm & 0.298 ± 0.046), and GEM/PEG-MWCNTs (35 ± 1.4 nm & 0.202 ± 0.074) respectively. The % entrapment efficiency was decreased GEM/HA-MWCNTs than GEM-MWCNTs (91.3 ± 1.7%) due to π-π interaction of MWCNTs and GEM after HA conjugation. The enhanced cell growth inhibition was observed in GEM/HA-PEG-MWCNTs (27 ± 1.1%) & GEM/HA-MWCNTs (22 ± 0.9%) was observed in HT29 cells. The hemolytic toxicity was comparatively less in GEM/HA-PEG-MWCNTs (7.73 ± 0.4%) when compared to free GEM (18.71 ± 0.44%). The anti-tumor study revealed that GEM/HA-PEG-MWCNTs significantly reduced tumor volume and increased survival. The improved pharmacokinetic parameter was observed in GEM/HA-MWCNTs and GEM/HA-PEG-MWCNTs treated group than in free GEM. The research team concluded that engineered MWCNTs as a safe and effective treatment for colon cancer therapy [86].

### Quantum dots

QDs are semiconductor NPs with a diameter of < 10 nm [87]. These are unique kinds of NPs because of their electrochemical, optical, physicochemical, and structural properties. Due to their sensing ability, they are widely used in biosensors, bioimaging, and drug delivery. These are also called fluorescent semiconductor inorganic NPs composed of groups II–VI, III–V, or IV elements [88]. QDs are classified based on their chemical composition, core type, core-shell, and alloyed QDs. The fluorescence imaging ability of QDs is because of their higher fluorescence output and photochemical stability [89]. QDs have taken attention in cancer therapy because of their unique physicochemical properties, and the drug can be selectively targeted into the tumor with less invasive techniques and without affecting normal cells [90]. The studies have reported that they are suitable for targeting colorectal tumors due to the exceptional properties of QDs, i.e., electrical conductivity, mechanical strength, thermal conductivity, optical, fluorescence emission, and excitation properties [91].

**Fig. 4:**
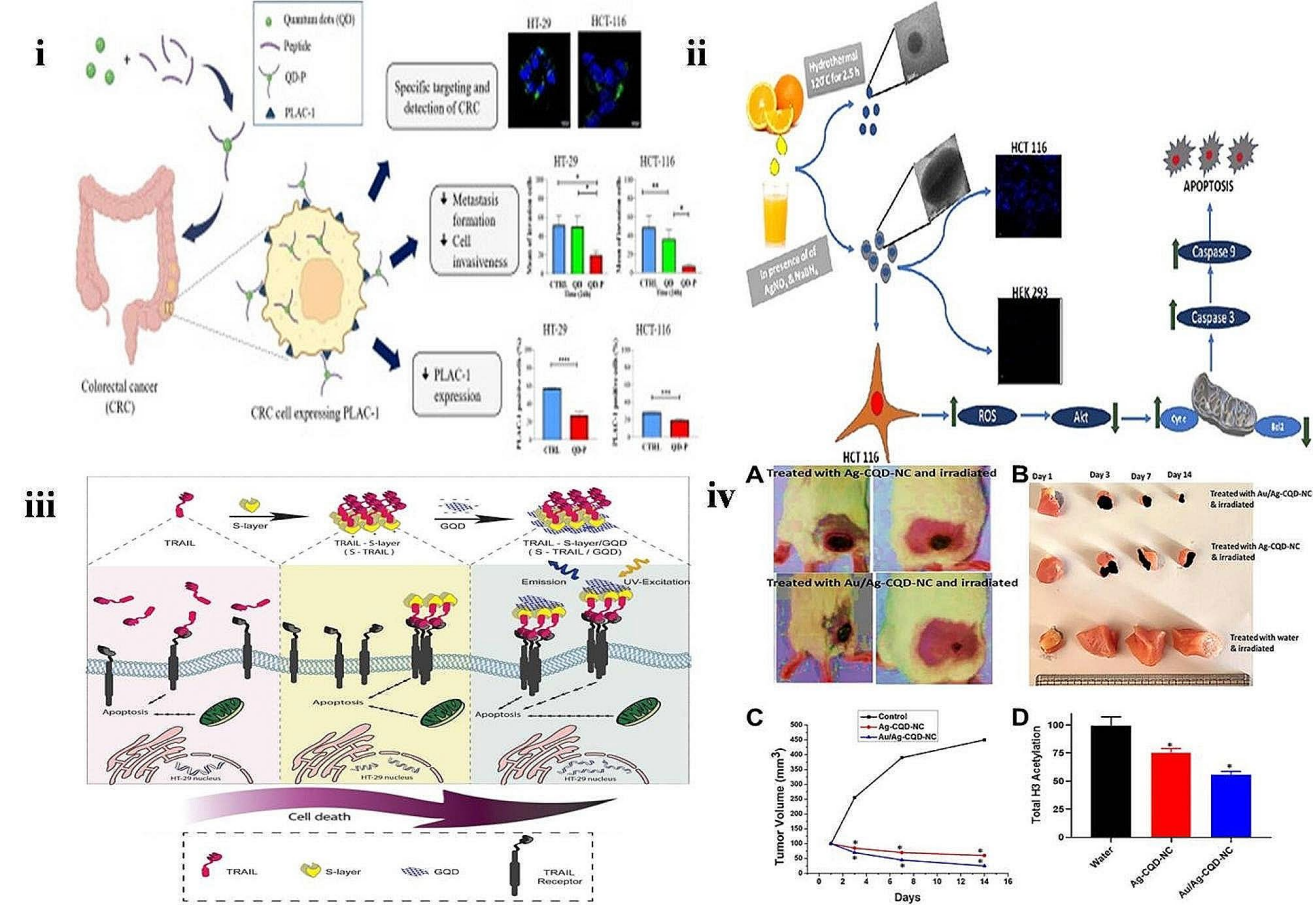
(**i**) Graphical representation of QD-P of graphene oxide as CRC theranostics, reproduced with permission from Ref. [93] (**ii**) Graphical representation of the development of CQD/Ag heterostructure from orange juice extract and characterization, reproduced with permission from Ref. [91]. (**iii**) Diagramatic illustration of S-TRAIL and S-TRAIL-GQD development and action on HT-29 cells, reproduced with permission from Ref. [98], (**iv**) Evaluation of tumor size (**A**) Tumor treated and irradiated with Ag-CQD-NC & Au/Ag-CQD-NC after with NIR laser, (**B**) Size of tumor treated and irradiated with Ag-CQD-NC, Au/Ag-CQD-NC with NIR laser, (**C**) Tumor volume (14 days) treated and irradiated with Ag-CQD-NC, Au/Ag-CQD-NC with NIR laser, (**D**) Total histone estimation in different groups. *p < 0.05, compared to normal, reproduced with permission from Ref. [100]

CRC is one of the most curable types of cancer if it is detected in an early stage. Chemotherapeutics are limited by their side effects and less responsible in an advanced stage. The currently available imaging techniques are limited due to a lack of diagnostic precision. Targeted NPs will be useful and bind to specific antigens to overcome this. Placenta-specific protein 1 (PLAC-1) is overexpressed in CRC [92]. Hence, Haider and teammates developed QDs of graphene oxide with a peptide named (GILGFVFTL) that has a high affinity towards (PLAC-1). The physicochemical and morphological characterization outcomes confirmed the coupling of QDs and peptides. Functionalized Q (QD-P) enhanced uptake in cells and cell cytotoxicity more than non-functionalized QDs. The QD-P toxicity downregulated the PLAC-I expression in HT-29 (53%) and HCT-116 (33%), respectively **(**Fig. 4**(i)).** The authors concluded that developed QD-P is a potential theranostic tool in detecting and treating colon carcinoma cells expressing the antigen PLAC-1protein [93]. In addition, biosensors have a significant role in the biomedical and pharmaceutical fields. Biosensors are sensing devices with unique characteristics like selectivity, sensitivity, rapid response time, etc. However, the limiting factor was a lack of sensitivity to low concentrations of cancer biomarkers. Combining nanotechnology and biosensors can partially overcome this issue [94]. Hence Pourakbari and colleagues attempted to develop a novel biosensing method for tungsten disulfide QDs (WS_2_-QD with electrochemical deposition of AuNPs for rapid and selective determination of c-Met proteins. Biosensor (M-13 Bacteriophage) is used as proof of concept to detect c-Met protein as a colon cancer biomarker. SEM studies showed size range of WS_2_-QD was 25 nm, and the average of the electrodeposited AuNPs was 25–45 nm. The outcomes revealed that the developed novel biosensor detected some concentration of c-Met protein in the colon cancer serum sample, which was unable to be seen by the ELISA kit. The authors concluded that the developed novel bioassay system has potential applications in the biomedical area [95].

Recently carbon-based QDs (CQD) emerged as a novel class of nanomaterials. Nowadays, naturally, available phytochemicals and secondary metabolites are generally used in cancer therapy because of their specificity and tissue-protective nature [96]. Based on this concept Mishra and colleagues developed a high fluorescence carbon QDs/Ag heterostructure from orange juice **(**Fig. 4**(ii))** and carried out in vitro anti-cancer activity using HCT-116 and HEK-293 cell line. Energy dispersive X-ray analysis confirmed the uniform distribution of carbon and Ag in their heterostructure. The carbon QDs of orange extract TEM images approved the uniform distribution of spherical-shaped particle particles without aggregation. The average diameter of particles was around 12 nm by Gaussian fitting and obtained around 200 particles. The carbon QDs/Ag heterostructure TEM images confirmed the uniform distribution of the spherical-shaped 60 particle’s average diameter was about 10 nm. In HCT-116 cells, higher concentration, cQDs/Ag induced 50% of cell death within 24 h. The HEK − 293 cell line showed 6 µg/ml induced negligible cell death [91]. Tumor necrosis factor (TNF)-related apoptosis-inducing ligand (TRAIL) is well known anti-cancer therapeutic agent. The use of TRAIL is limited due to the weak pharmacokinetic profile leading to poor circulation and a short half-life resulting in fast renal excretion. Hence it may lead to insufficient availability of TRAIL to the targeted site and also resistance in cancer cells [97]. To address this issue, Lotfollahzadeh and co-workers developed a novel TRAIL-S-layer (S-TRAIL) fusion protein that was coupled to QDs of graphene(GQDs) via noncovalent interactions on colon cancer cells **(**Fig. 4**(iii))**. TEM results of S-TRAIL, GQD, and the S-TRAIL/GQD revealed that GQDs were in narrow size distribution with average diameters of 2.01 ± 0.28 nm, S-TRAIL exhibited as amorphous aggregates and S-TRAIL/GQDs appeared as a spherical shape with an average diameter of 17.77 ± 2.03 nm. The anti-cancer efficacy in HT-29 by MTT and flow cytometry assay revealed that 80% of cell death. The authors concluded that the developed novel S-TRAIL/GQD complex is a promising nanohybrid technique against colon cancer [98]. PDT emerged as the best treatment technique for various cancers. It utilizes laser light and radiation to kill the cancerous cells. However, this procedure is restricted due to the precision of the imaging technique. Hence to enhance the efficacy, the treatment system should facilitate the imaging techniques [99]. To address this, Liu and colleagues produced Au and Ag-doped CQD nanohybrid composite for effective PDT against colon cancer cells. TEM studies showed that developed Au/Ag-CQD-NC exhibited a quasi-spherical shape with a homogenous distribution. Energy dispersive X-Ray spectra confirmed the presence of C, O, N, Ag, Au, and Cu. This demonstrated the successful preparation of Au/Ag-CQD-NC. The cell viability was 88% upon treatment with Au/Ag-CQD-NC (100 µg/ml) and 85% with Au/Ag-CQD-NC (4000 µg/ml). This indicated prepared novel nanocomposite was not cytotoxic. In vivo studies revealed that after irradiation with NIR and injection of Au/Ag-CQD-NC, the tumor size reduced to small and turned black **(**Fig. 4**(iv))**. Hence authors concluded that Hybrid nanocomposite has advantages over medical conditions [100].

### Mesoporous silica nanoparticles

**Fig. 5:**
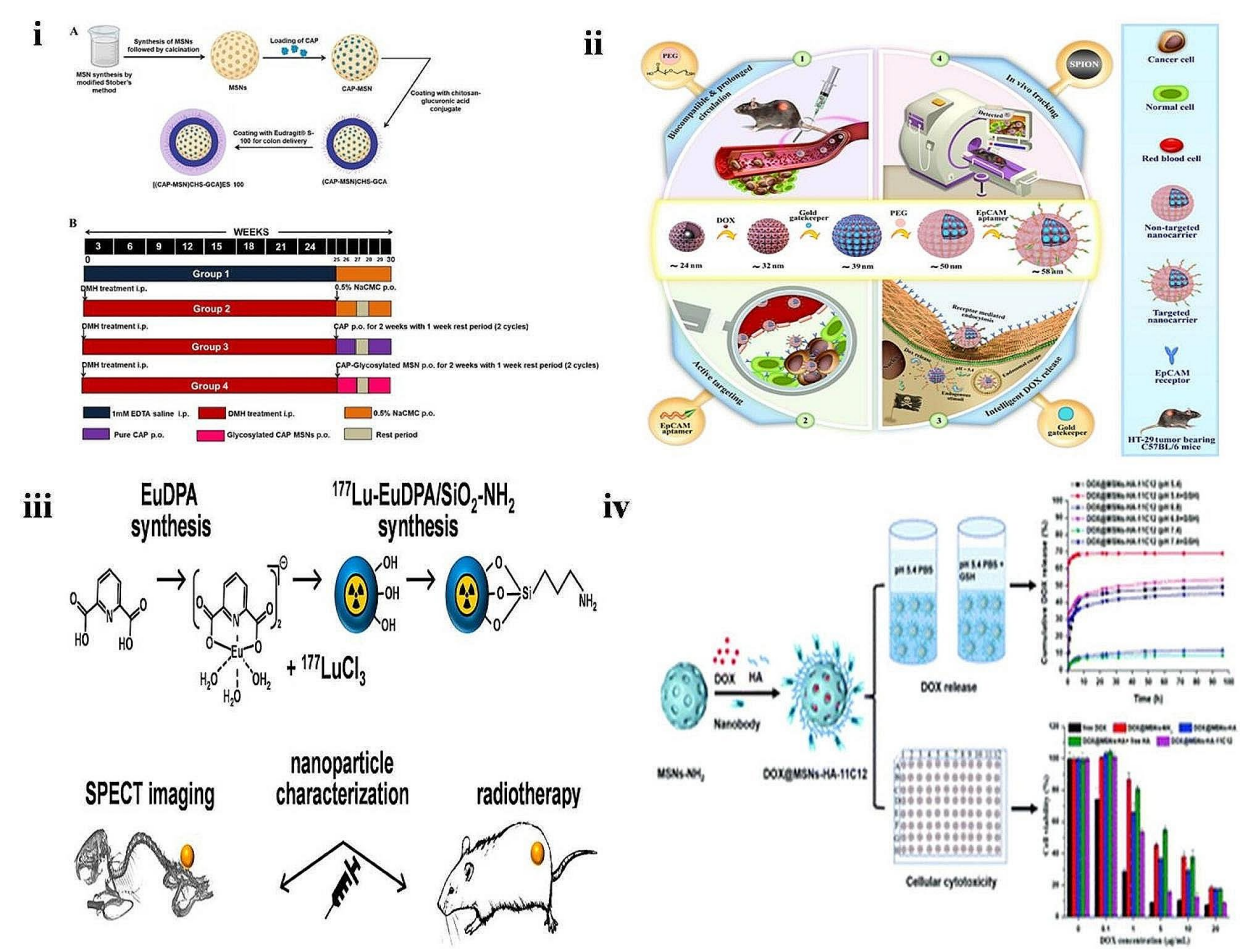
(**i**) Representation of study protocol (**A**) Synthesis of MSNPs, (**B**) Treatment protocol reproduced with permission from Ref. [105]. (**ii**) Graphical representation of the development of magnetic MSNPs, reproduced with permission from Ref. [107]. (**iii**) Graphical representation of the development of ^177^Lu-Eu-DPA/SiO2-NH2 NPs for CRC, reproduced with permission from Ref. [110]. (**iv**) Diagrammatic illustration of nanobody conjugated HA MSNPs reproduced with permission from Ref. [112]

MSNs are a porous, solid framework that chemically has the honeycomb-like structure of silica (SiO_2_). They have several advantages, like the tunable size of particles, uniform porous structure, high surface area, and good biocompatibility. There are three types of MSNs (i) Ordered type MSNs, (ii) hollow type MSNs, and (iii) core type MSNs [101, 102]. The therapeutic agent loads into the core or to the surface of MSNs by covalent binding and electrostatic adsorption. The mesoporous form of silica has the potential to load high quantities of the drug in its core; with low toxicity and subsequent drug release, these unique features make them suitable for targeted drug delivery systems. These are stable NPs because of the strong bond between the Si-O [103]. Studies reported that MSNs induced endocytosis mechanisms in different mammalian cancer cells [104]. Conventional treatment for CRC is ineffective because the drug’s desired concentration has not reached the targeted site. Also, several studies have reported drug-related toxicities due to a lack of site-specificity. To overcome this, Narayan and teammates developed Chitosan glucuronic acid conjugated pH-responsive MSNs of capecitabine to target CRC **(**Fig. 5**(i)).** The study showed that developed MSNs exhibit average pore diameter and particle sizes of 8.12 ± 0.43 nm and 245.24 ± 5.75 nm with a pore volume of 0 0.73 ± 0.21 cm^3^/g. The high drug loading (180.51 ± 5.23 mg/g) was reported for larger pore volume MSNs. The drug release was around 85.16 ± 1.45% at acidic pH (5.5) within 72 h. The high uptake of the developed novel system was observed in HCT 116 cells. In vivo, study outcomes revealed that developed glycosylated MSNs reduced the tumors, aberrant crypt foci, inflammation, and toxicity. The authors concluded that the promising outcome from developed NPs can be used as an effective carrier against CRC [105]. The developing novel system should consider the physicochemical properties of NPs and tumor microenvironment features to upgrade the drawbacks associated with conventional therapy and enhance therapeutic efficacy. Studies reported that including traceable imaging systems and therapeutic agents in single nano delivery systems develops a better theranostic action plan, resulting in the most efficient targeted delivery through image-guided delivery [106]. So, Iranpour and teammates developed a novel system of magnetic MSNs as a smart and targeted drug delivery system for CRC therapy. Developed NPs exhibited a mean final size diameter of 58.22 ± 8.54 nm. SEM studies confirmed that developed nanocarriers had a uniform and spherical morphology. TEM studies confirmed the porous structure of nanocarriers. In the EpCAM-positive HT-29 cells, higher cytotoxicity, and cellular uptake of nanocarriers were observed than in EpCAM-negative CHO cells. In vivo studies in a mouse model showed that targeted nanocarriers could effectively doxorubicin accumulation in the tumor area, inhibit its growth, and ultimately reduce the adverse side effects **(**Fig. 5**(ii))** [107]. Epithelial cell adhesion molecule (EpCAM) is a transmembrane glycoprotein, and they have a vital role in cell signaling, migration, proliferation, and differentiation. Several studies reported that this biomarker is associated with CRC metastasis; hence this can be considered an excellent therapeutic agent for CRC treatment. Considering this, Mosawi and colleagues developed non-targeted PEGylated superparamagnetic iron oxide NPs (SPION) conjugated MSNs for the controlled release of 5-FU. The developed nanocarriers exhibited a diameter of 78 nm. The surface area of SPION-MSNs was found to be 636 m^2^g^− 1^. The drug release was initially rapid, and sustained release was up to 96 h at pH5.4. In vitro studies using HT-29 cells proved that cellular uptake of developed nanocarrier was at a high rate. The study revealed that the targeted SPION-MSNs significantly reduced tumor growth in C57BL/6 mice bearing HT-29 tumors compared to the injection of free 5-FU. The authors concluded that the developed nanocarrier is a potential theranostic platform for Ep-CAM-positive CRC [108]. CRC is usually diagnosed at the last stage; hence radiotherapy emerged as an enormous treatment approach. Studies have shown that to enhance the therapeutic efficacy of radiotherapy using radionuclides, the process of nanotechnology can be used effectively by using multifunctional nanomaterials [109]. Hence Viana and his team developed ^177^Lu labeled-Eu doped pyridine 2, 6-dicarboxylic acid (DPA) designed into ^177^Lu-Eu-DPA/SiO2-NH2 functionalized hybrid NPs **(**Fig. 5**(iii))** SEM studies of ^177^Lu-Eu-DPA/SiO2-NH2 functionalized hybrid NPs exhibited small blocks of irregular patterns. TEM studies confirmed small particles having a size of less than 20 nm. DLS study showed an average particle size of 21.7 nm for silica NPs and 63.3 nm for ^177^Lu-Eu-DPA/SiO2-NH2. The loading of ^177^Lu into Eu-DPA/SiO2-NH2 was efficient, and up to 93% of the radioactivity resulted from the final compound. The SPECT/CT image analysis confirmed the localization of the tumor was maintained up to 48 h after intratumoral administration. The tumor growth was significantly reduced after treatment of ^177^Lu-Eu-DPA/SiO2-NH2 NPs. The authors confirmed that it is a promising agent for future development in the clinical treatment of CRC [110]. MSNs can achieve controlled delivery of drugs into CRC cells, which can be boosted by switching to the gatekeeper modifications like stimuli sensitive exhibits system more advantages [111]. Hence Fan and co-workers developed CEA/CD44 targeting nanobody (11C12)-conjugated HA-modified doxorubicin MSNs (DOX@MSNs-HA-11C12) with pH and redox sensitivity **(**Fig. 5**(iv))**. TEM study results showed that the average particle size of MSNs-NH_2_ was around 144.9 ± 5.2 nm, which is barely changed with DOX@MSNs-HA-11C12. The in vitro drug release study showed that the release of drug rate accelerated in a weak acidic environment pH [pH 7.4 (GSH) + pH 6.8 (GSH) + pH 5.4 (GSH)] and the presence of glutathione. In vitro, cytotoxicity study showed good biocompatibility, safety, and DOX@MSNs-HA-11C12) led to higher cell apoptosis in LoVo CRC. The authors concluded that developed dual CEA/CD44 formulation can promote more accurate drug delivery to CRC cells [112].

### Iron oxide nanoparticles

On Earth, iron is abundantly available in the form of iron oxides. The synthesis of multifunctional magnetic NPs is widely preferred and has enormous applications as a contrasting agent in MRI [113]. These have wide applications in the biomedical field, which include molecular imaging, hyperthermia treatment techniques, therapeutic agent delivery, etc. This system delivers the drug directly into the cytoplasm through the endocytosis mechanism. It mainly avoids the pump transporter recognition in multi-drug resistance cancer cells [114]. The dual ability of Fe_3_O_4_NPs, i.e., its magnetic and photothermal properties, made it suitable for cancer therapy [115].

**Fig. 6:**
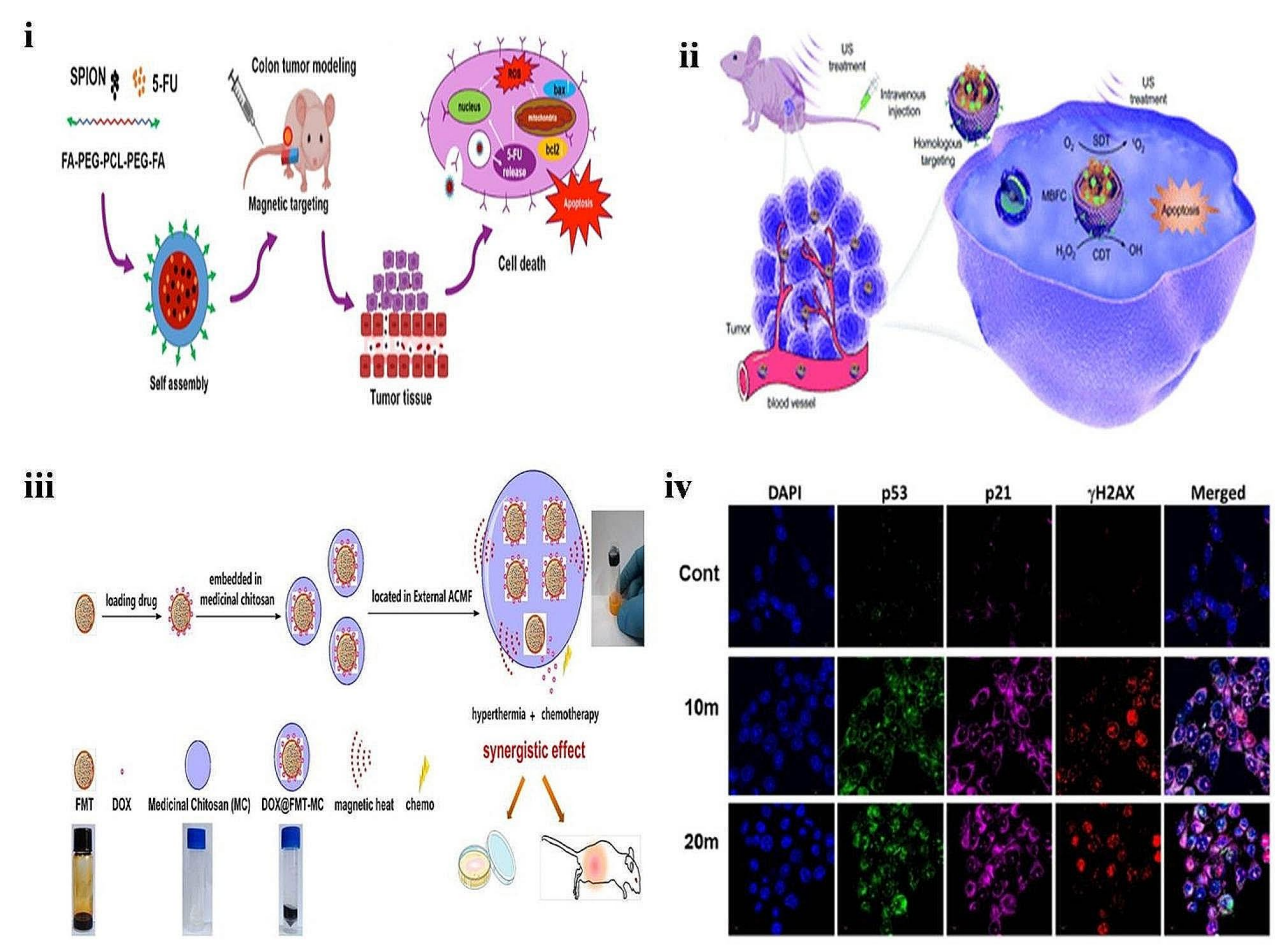
(**i**) Graphical representation of folic acid conjugated 5-FU-PEG-PCL-PEG-FA mechanism of action on colon cancer cells, reproduced with permission from Ref. [117]. (**ii**) Graphical representation of biomimetic sonosensitizer targeted iron oxide nanocrystals, reproduced with permission from Ref. [118]. (**iii**) Graphical representation of the synthesis of DOX-ferumoxytol iron oxide nanoparticles, reproduced with permission from Ref. [119]. (**iv**) Expression of p53, p21, and cH2AX upon light exposure in treated HCT 116, reproduced with permission from Ref. [121]

The studies have shown that the approach of conjugated NPs with ligands exhibited the promised outcomes in targeted drug delivery systems [116]. So, Mirzaghavami and teammates synthesized a triblock copolymer (PEG-PCL-PEG), 5-FU loaded magnetite NPs conjugation within folic acid as a ligand (5-FU-PEG-PCL-PEG-FA) **(**Fig. 6**(i))**. The TEM studies exhibited the spherical shape of particles with a hydrodynamic diameter of 85 nm. The release studies showed that drug release is dependent on pH with a %CDR of 23% at 24 h. The uptake studies revealed the uptake of 5-FU-PEG-PCL-PEG-FA-NPs colon carcinoma cells (HT-29) than in normal endothelial cells (HUVEC). The resulting novel system exhibited enhanced anti-tumor efficiency, high mouse model survival rate, and tumor inhibitory volume. The authors concluded that a developed novel system could potentially treat colon cancer [117]. Similarly, Chen and colleagues developed iron oxide nanocrystals with sonosensitizer conjugated as a combination of chemodynamic (CDT) and sonodynamic therapy (SDT) against CRC **(**Fig. 6**(ii))**. The Fe_3_O_4_ nanoparticles were synthesized using a basic co-precipitation method using bovine serum albumin conjugation with Ce6. The resulting nanocrystals exhibited strong catalytic ability and highly efficient sonodynamic features. The cellular uptake studies I CT 26 tumor cells resulted in a high uptake of nanocrystals via a homologous targeting mechanism. The developed nanocrystals combination with SDT and CDT induced apoptosis of cells in vitro and significantly inhibited growth of tumor in tested mice model. The authors concluded that biomimetic nanocrystals could be a potential approach for CRC tumor targets [118].

Iron-based nanomedicine system that is the principal representative of INPs approved and widely applicable in the biomedical field. So, Chen and teammates designed DOX ferumoxytol Chitosan-based iron oxide nano hydrogel (DOX-FMT-CS-Fe_3_O_4_-HYD) as chemotherapy in HT-29 cells **(**Fig. 6**(iii))**. The resulting DOX-FMT-CS-Fe_3_O_4_-HYD with an angular frequency of 3.5 rad/s loss and storage modulus cross with equal modulus. The release study showed that DOX was released from the HYD, and FMT-embedded hydrogel generated heat. The content of leaked iron concentration was increased over time (In 16 h, 7.6 to 9.2%, and 40 h 89–98%). Cell apoptosis assay showed synergetic efficacy with 32.4% on HT 29 cells. Xenografted mice tumor model exhibited better heating performance from the DOX-FMT-CS-Fe_2_O_3_-HYD complex [119]. Recently PDT (PDT) has been widely used in CRC because it causes less toxicity to normal healthy cells. However, this treatment system has limitations like tumor selectivity, shorter ROS lifetime, and less laser penetrability. Studies have shown that this problem can be overcome using a metal NP hybrid system [120]. Hence Alkahtane and co-workers developed oleic acid (OA) CS-coated Fe_3_O_4_ NPs (OA-C- Fe_3_O_4−_NPs) against CRC carcinoma. TEM studies exhibited that OA-C- Fe_3_O_4−_NPs had a network-like appearance with a segment length of 300–500 nm and a width of 350–450 nm. The morphology was found like nano egg-like properties with a size of 15 nm and a width of 5 nm. The study outcomes revealed that the light-induced enhanced ROS activity of the OA-C- Fe_3_O_4_NPs led to cell death *via* activating caspase 9/3 **(**Fig. 6**(iv)).** The authors concluded that in vitro MRI experiments in HCT 116 cells showed that the nano-hybrid system is a highly therapeutic MRI contrasting agent for diagnosing CRC [121]. Several studies reported that combinational therapy suppresses tumor growth [119]. So, Dabaghi and co-workers formulated functionalized CS-coated magnetic NPs of 5-FU (5-FU-MNPs) and termed this combination therapy “thermo-chemotherapy.” The developed 5-FU-MNPs exhibited a mean diameter of 176 ± 7 nm, followed by PDI of 0.17 ± 0.01 and the ζ-potential of − 27.9 ± 0.5 mV. The developed thermo-chemotherapy resulted in distinct tumor regression. The authors concluded that thermo-chemotherapy induces thrombogenic collagen fiber leading to an imbalance in nutrient supply to the tumor cells and cell death [122].

### Gold nanoparticles

AuNPs are wine-red compounds with antioxidant properties, and the size varies from 1 nm to 1000 nm. It has distinct forms like spherical, sub-octahedral, irregular shapes, nanorods, nanotriangles, etc. [123]. Au is a non-reactive chemical element and this widely used due to its occurrence, easy handling, simple fabrication, easy modification, corrosive resistance, chemical stability, biocompatibility, etc. [124]. AuNPs are widely applicable in the biomedical field due to their small size, stability, low toxicity, optical properties, bioimaging, and large surface area [125]. AuNPs absorb incidence photons and convert them into heat; hence generated heat destroys the cancer cells. Different types of AuNPs include Au-nanorods, Au-nanostars, and Au-nanocages [126].

PTT is an effective treatment for CRC condition. This therapy is mainly based on photosensitizers. The irradiation of photosensitizers with NIR 808 nm leads to the activation of photosensitizers. AuNPs efficiently convert high light into heat; hence, they are widely used as photothermal agents in PTT. So, Costantini et al. synthesized highly photostable branched AuNPs (B-AuNPs) to eradicate colon cancer cells. The synthesized B-AuNPs exhibited an average particle size of 180 ± 10 nm. The cell granularity was enhanced 1.74-fold in B-AuNP cells compared to unexposed cells. This confirmed the uptake of B-AuNPs by colon cells. The treated colon cancer cells resulted in a significant decrease in cell viability. The authors concluded that the synthesized photostable B-AuNPs could target colon cancer cells [127].

**Fig. 7:**
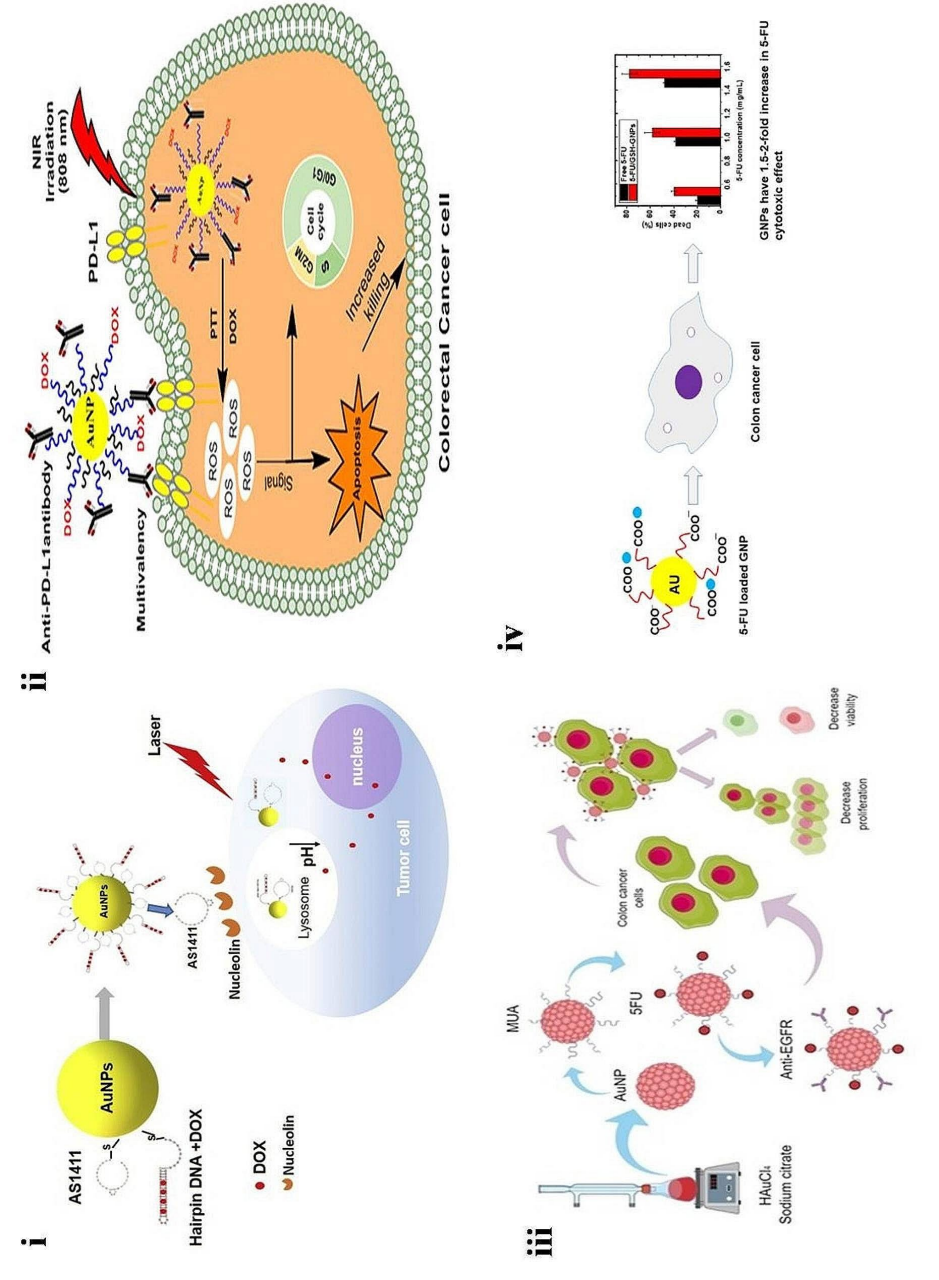
(**i**) Diagrammatic representation of chemophothermal effect of AuNPs on colon cancer cells, reproduced with permission from Ref. [128]. (**ii**) Graphical representation of PD-L1-AuNPs-DOX targeting colon cancer cells, reproduced with permission from Ref. [130]. (**iii**) Diagrammatic illustration of the effect of AuNPs- 5FU-EGFR on colon cancer cells, reproduced with permission from Ref. [132]. (**iv**) Graphical illustration of 5FU-AuNPs inducing cell death in CRC cells, reproduced with permission from Ref. [133]

Similarly, Zhang and colleagues developed laser-triggered AuNPs of doxorubicin modified by AS1411 and DNA rich of intercalation **(**Fig. 7**(i))**. TEM images confirmed that the diameter of NPs increased after the modification of AuNPs with DNA and DOX. The average size of 399.5 nm with PDI 0641 and ζ–potential − 18.9 mV. The developed system bound toward SW480 colon cancer cells and enhanced the uptake of NPs in vitro. The AS1411-based AuNPs increased the cytotoxicity by inhibiting cell proliferation in SW480 colon cancer cells under laser exposure. They concluded that the developed study emerged as a novel alternative platform with targeted PTT against colon cancer cells [128]. Studies have shown that survival of CRC is significantly associated with the overexpression of programmed cell death ligand (PDL1). Hence developing a system targeting PDL1 may enhance the survival rate [129]. So, Emami and teammates developed AuNPs of DOX and anti-PD-L1 antibody (PD-L1-AuNPs-DOX) for CRC photochemotherapy **(**Fig. 7**(ii))**. The developed PD-L1-AuNPs-DOX exhibited an average particle size of 40.0 ± 3.1 nm. The Intracellular uptake of DOX increased in CT-26 cells. Due to enhanced uptake, the apoptosis of cells was around 66% in CT-26 cells. The association of PD-L1-AuNPs-DOX with irradiation of NIR leads to cell cycle arrest and cell apoptosis in CT-26 cells. The authors concluded that PD-L1-AuNPs-DOX, in association with PTT, acts as a synergistic treatment for localizing the CRC [130]. The studies showed that CRC cells overexpressed EGFRs. Hence targeting this receptor may be targeted therapy to treat CRC [131]. Therefore, Liszbinski and co-workers developed 5-FU-AuNPs coated with anti-EGFR antibodies **(**Fig. 7**(iii))**. The developed AuNPs-5FU-anti-EGFR exhibited a particle size of 18.83 ± 1.52 nm, followed by a ζ–potential of -33.1 ± 3.78 mV. TEM results confirmed the presence of spherical-shaped particles. The developed novel system induced cell death in HT-29 and HCT-116 cells. The AuNPs-5FU-anti-EGFR enhanced cell apoptosis and impaired cell proliferation in both cell lines. The authors concluded that functionalized AuNPs with monoclonal antibodies potentially delivered 5-FU to colon cancer cells and could be a novel strategy against CRC [132]. The 5-FU is a well-known chemotherapeutic agent against CRC. However, clinical use of this is hindered by several side effects. Hence to overcome these side effects and enhance the anti-cancer efficacy, it is loaded with the AuNPs using two thiol-containing ligands, namely thioglycolic acid (TGA) and glutathione, by Safwat and colleagues **(**Fig. 7**(iv))**. The resulting NPs exhibited a size of ∼9–17 nm. The high loading of 5-FU was observed with a ligand molar ratio of 1:1 for TGA-GNPs and 2:1 for GSH-GNPs, respectively. The drug release from AuNPs was pH-dependent. The developed 5-FU/GSH-AuNPs inhibited cell apoptosis and cycle progression. The study also revealed that the developed novel system exhibited a 2-fold higher anti-cancer effect when compared with free 5-FU. The authors concluded that developed AuNPs potential to enhance 5-FU anti-cancer efficacy in colon cancer cells [133].

### Silver nanoparticles

AgNPs are widely used in the industry, food, and medical fields due to their distinctive physical and chemical properties. Due to its broad spectrum of anti-microbial properties, it is used in the manufacture of household products, industries, health care products, pharmaceutical industries, etc. [134]. The AgNPs have anti-cancer properties and have a mechanism of action that releases Ag + ions from AgNPs enters into the cell and reach the mitochondria and interact with the thiol groups. After interaction binds to the NADPH dehydrogenase enzymes and releases the ROS, this reacts with respiratory enzymes and blocks ATP synthesis. Also, the formed ROS binds to DNA and RNA, hindering the cell replication and synthesis of proteins resulting in cell death [135]. So, AgNPs are widely used as an anti-cancer drug carrier to the tumor site [136].

**Fig. 8:**
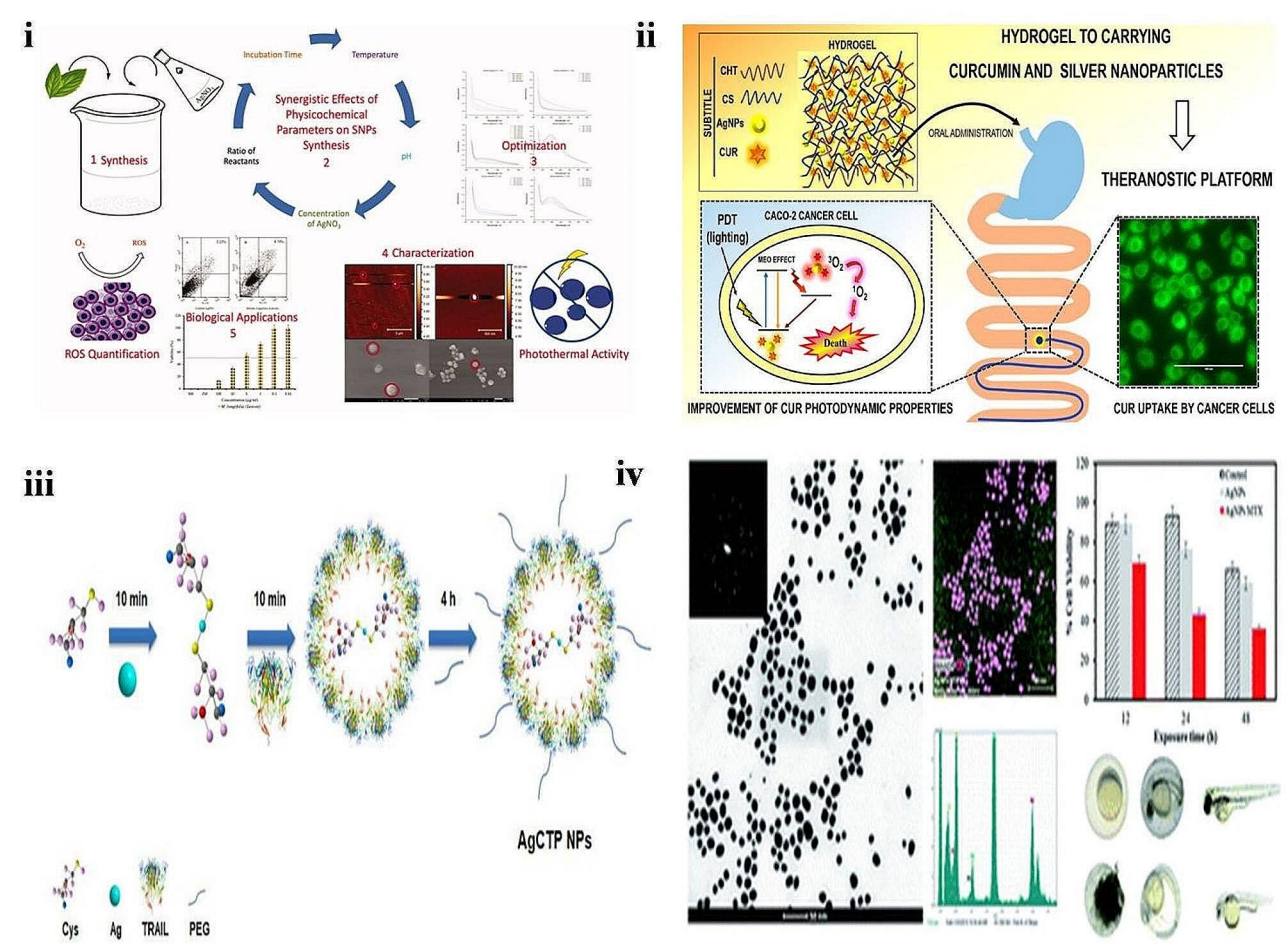
(**i**) Graphical representation of biogenesis of silver nanoparticles, reproduced with permission from Ref. [138]. (**ii**) Graphical representation of hydrogel containing curcumin and AgNPs, reproduced with permission from Ref. [139]. (**iii**) Schematic representation of the synthesis of AgCTP nanoparticles, reproduced with permission from Ref. [142]. (**iv**) Schematic representation of AgNPs-MTX against colon and lung cancer cells, reproduced with permission from Ref. [143]

Recently green nanotechnology emerged as a new approach that uses various metal NPs for targeted drug delivery systems. Studies have shown that Ag is a metal that has a strong cytotoxic effect against cancer cells; it is widely used in the biomedical field [137]. So, Mashwani and teammates developed AgNPs of Mentha longifolia leaves aqueous extract and evaluated their action against colon cancer cells **(**Fig. 8**(i))**. Spectrophotometric analysis exhibited that 120 °C with acidic pH 3mM of AgNO_3_ when mixed in a 1:9 ratio (plant extract: AgNO_3_) optimized condition for the synthesis of AgNPs. The studies showed that NPs are spherical and have an average size in the 10–100 nm range. The AgNP concentration of ∼10 µg/ml destructed population of ∼66% of Leishmania and 8.73 µg/ml was selected as IC_50_. Cell apoptosis assay on HCT 116 cells showed plant extract and AgNPs not active against colon cancer cells. The AgNPs of Mentha longifolia revealed that they can generate free radicals and not result in any photothermal activity [138]. Several studies reported curcumin efficacy against CRC, and clinical studies reported its poor bioavailability and low absorption with rapid metabolism. Hence Freitas and colleagues developed a multimodal platform of incorporating curcumin and AgNPs into polysaccharide-based hydrogels to enhance the photodynamic effect of curcumin via the metal-enhanced singlet oxygen effect **(**Fig. 8**(ii))**. The resulting NPs exhibited an average diameter of 4.1 nm. HR-TEM analysis confirmed that the particles are spherical with uniform distribution. The chitosan and chondroitin sulfate hydrogel containing curcumin AgNPs exhibited a homogenous macrostructure with rough surface and excellent miscibility and stability. Cellular study results revealed that developed hydrogel containing curcumin AgNPs is safe for healthy cells. The PDT selective illumination study showed that developed AgNPs inhibited Caco-2 colon cancer cells. The authors concluded that curcumin could be an efficient diagnostic fluorescent probe in the theranostic system [139]. In another investigation, Lu and his team developed reusable composite graphene and AgNPs (GO-AgNPs) for the catalytic reduction of 4-nitrophenol and as an efficient treatment of CRC. FE-SEM images revealed that small quantities of AgNPs were distributed on the surface of GO in the developed system. EDX analysis confirmed that the developed nanocomposite comprises Ag, C, and O elements. TEM images confirmed that GO-AgNPs were uniformly distributed with a narrow size distribution (5 nm). The developed GO-AgNPs showed significant cell death in CRC cell lines (HT-29, HCT 116, HCT-8 [HRT-18], and Ramos.2G6.4C10). The high percentage of cell apoptosis results were exhibited in HT-29 cells. The antioxidant study revealed that GO-AgNPs inhibited 50% of the DPPH molecules in 344 mg/mL concentration. The authors concluded that GO-AgNPs could be a chemotherapeutic supplement for CRC [140]. Several studies reported that TRAIL could induce cell death, but the use of this is limited due to its short half-life and less stability. Due to this issue, TRAIL could not reach the tumor site [141]. So, Birtekocak and co-workers synthesized TRAIL-conjugated PEGylated AgNPs (AgCTP-NPs) against colon cancer cells **(**Fig. 8**(iii))**. The synthesized AgCTP-NPs were light brown and had good water solubility. The average particle size was around 128.12 ± 8.02 nm, and the ζ–potential of -28.75 ± 2.02 mV. The AgCTP-NPs showed a significant decrease in HT-29 cell colonies and confirmed the upregulation of apoptotic proteins. The authors concluded that AgNPs could be a good vehicle for TRAIL therapeutic proteins, which can be used to treat colon tumors [142].

Similarly, Rozalen and colleagues synthesized PEGylated AgNPs of methotrexate embedded in GO (AgNPs-MTX) **(**Fig. 8**(iv))**. The developed AgNPs-MTX exhibited a spherical shape of NPs with an average size of 13 nm with the polydisperse size of particles with a wide distribution range of 7–21 nm. In vitro drug release profile results revealed that 77–85% of MTX is released from AgNPs. The release profile was fitted to the Higuchi model and followed the diffusion mechanism. The AgNPs-MTX significantly reduced HCT-116 cells, and the reduced IC_50_ from 186 to 63 µg mL^− 1^ was observed after 12 and 48 h of exposure. The authors concluded that a synergetic effect was observed when MTX was conjugated with AgNPs [143].

### Zinc oxide nanoparticles

Zinc is essential for various human body parts, like muscles, bones, skin, etc. Recently, ZnONPs emerged as an outstanding tool in optical, electrical, and biomedical research [144]. Studies have been shown that ZnONPs widely used in cancer therapeutics due to their cancer cell-specific toxicity. ZnONPs lead to cancer cell death via a pH-dependent mechanism. The Zn^2+^ dissolves in low pH and generates ROS, leading to cancer cell death [145]. ZnONPs have biomedical applications like bioimaging, targeted drug delivery systems, and tissue engineering. These are also anti-oxidant, anti-diabetic, and anti-microbial agents [146].

**Fig. 9:**
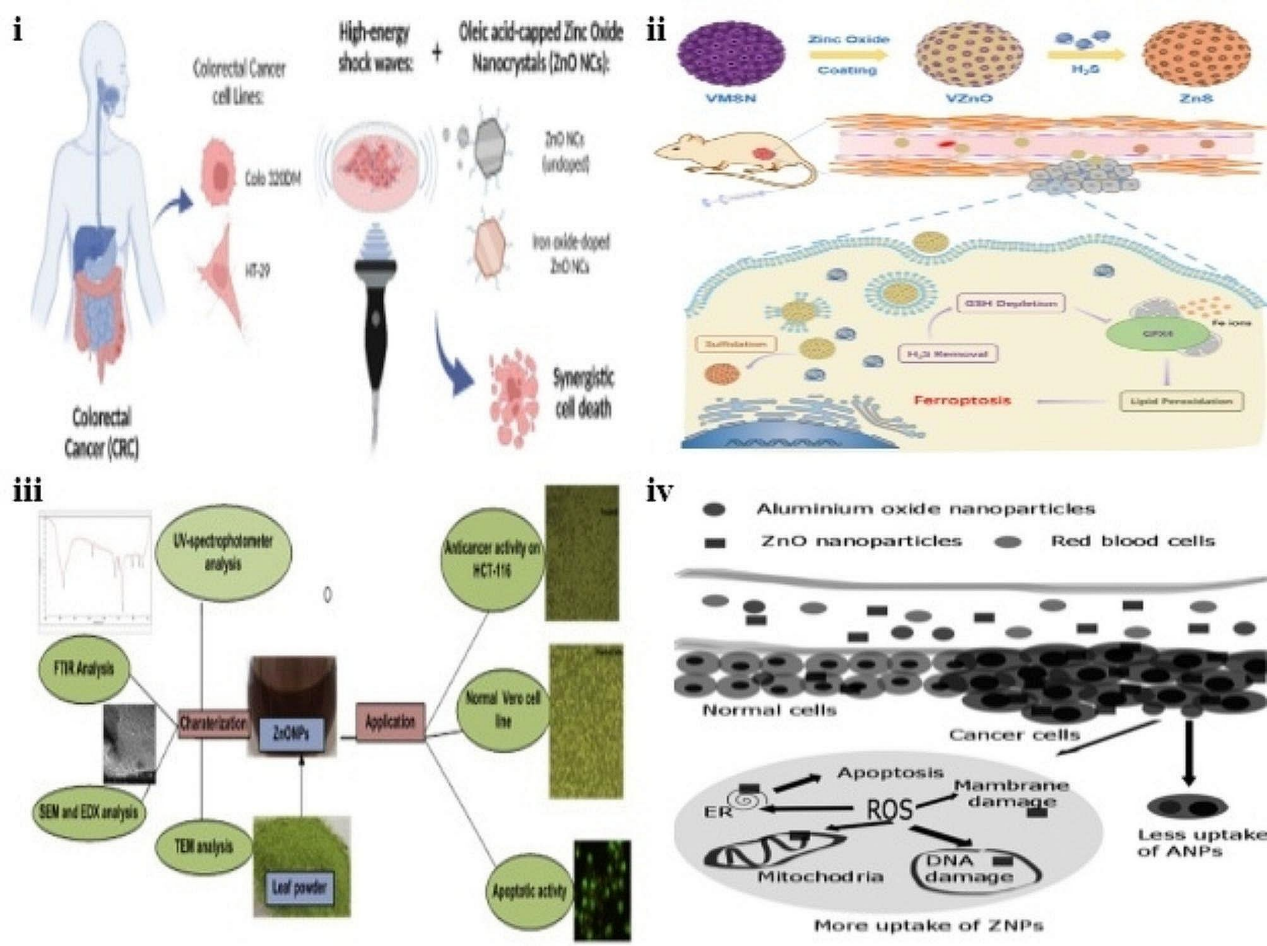
(**i**) Graphical representation of the synergetic effect of ZnO-nanocrystals and shock waves on CRC, reproduced with permission from Ref. [147]. (**ii**) Schematic illustration of the mechanism of ZnO nanospheres in H_2_S scavenging and ferroptosis in CRC, reproduced with permission from Ref. [149]. (**iii**) Schematic representation of the synthesis of ZnO-NPs and its evaluation in HCT-116 cell line, reproduced with permission from Ref. [151]. (**iv**) Graphical representation of anti-proliferative effects of ZnO-NPs and Al(OH)_3_-NPs on HT-29 cells, reproduced with permission from Ref. [152]

Recently, Racca and colleagues developed an NP’s (oleic acid-Zn oxide nanocrystals) anti-tumor effect with external high-energy shock waves as a combinational therapy against CRC. The undoped ZnO nanocrystals (ZnONC) and iron (Fe) doped with oleic acid and aminopropyl functionalization ensure the colloidal stability of nanocrystals. The FE-SEM images revealed that both the nanocrystals are in a unique shape with average particle diameters of 4 and 10 nm. HR-TEM analysis revealed that Fe-doped and undoped nanocrystals were exhibited as single crystals in a spherical shape. The cell lines studies showed that both the nanocrystals had good bio-tolerance and rate of internalization in HT-29 and Duke’s type C Colo 320-DM cells. Cell cytotoxicity studies showed that combined therapy boosted anti-tumor activity in Colo 320-DM cells more than in control groups. The continued shock wave treatment thrice per day further resulted in the mortality of CRC cells **(**Fig. 9**(i))**. The authors concluded that combination therapy enhanced the anti-tumor efficacy of CRC cells [147]. Studies have reported that the upregulation of hydrogen sulfide (H_2_S) aids the growth and progression of colorectal tumors. Hence, removing H_2_S can be a significant step in treating CRC [148]. So, Pan and co-workers developed the H_2_S-responsive ZnO nanosphere coated with virus-like silica (VZnO) for CRC therapy **(**Fig. 9**(ii))**. The virus-like formation MSNs exhibited a mono-distributed average particle size of ≈ 120 nm. The elemental mapping analysis showed the distribution of Si and O elements in ZnONPs. The H_2_S scavenging study using HCT-116 and CT 26 cells resulted in a reduction in glutathione levels as well as cell viability within 24 h. TEM images revealed that the treatment with VZnO led to an abnormality in mitochondria of malignant epithelial cells with shrinkage and disappearance. In vivo biodistribution study in Balb/c mice model showed IV injections of VZnO@fluroscence isothiocyanate signals were detected within 30 min and reached peak level within 4 h after injection. The authors concluded that H_2_S scavenging-induced ferroptosis strategy using VZnONPs could be a promising therapy for CRC [149]. Ye and teammates developed ZnONPs in oxaliplatin-resistant CRC cells in another investigation. The developed ZnONPs reduced the viability of HCT116 and HCT8 cells. Also, ZnONPs reduced the viability of oxaliplatin resistance HCT-116 and HCT8 cells. Also, this study revealed intensified cell proliferation after hindrance of miR-1321 or overexpression of HIF-2a compared with the ZnONPs treated cells.

The administration of ZnONPs suppressed oxaliplatin resistance HCT116 cell-induced tumors in the Balb/C mice model. The authors concluded that ZnONPs are the effective treatment technique against chemoresistant CRC [150]. In another investigation, Shahanaz and colleagues synthesized the ZnONPs of the aqueous extract of leaves of Artocarpus heterophyllus **(**Fig. 9**(iii))**. The SEM analysis exhibited roughness of developed ZnONPs. TEM images confirmed the well-dispersed spherical shape of particles in the size range of 12–24 nm. The synthesized ZnONPs of the extract resulted in a significant cytotoxic effect on the colon cancer cell line (HCT-116). AO/EB staining revealed that ZnONPs treated HCT-116 cells characterized by nuclear condensation, fragmentation, and cell apoptosis. The authors concluded that ZnONPs of Artocarpus heterophyllus act as an excellent anti-cancer agent in colorectal adenocarcinoma cell lines [151].

Similarly, another team evaluated the anti-proliferation effect of ZnONPs and Aluminium hydroxide [Al(OH)_3_] NPs on colon cancer cell lines (HT-29). The resulting NPs exhibited an average particle size of 50.89 nm. TEM images confirmed that both Al(OH)_3_-NPs and ZnONPs revealed high homogenous surfaces of particles. The developed ZnONPs significantly inhibited cell proliferation in a dose-dependent manner **(**Fig. 9**(iv))**. The cell viability in HT-29 cells treated with 50 and 100 µg/ml of ZnONPs showed 49% & 33% and Al(OH)_3_-NPs 65% and 41%, respectively. The authors concluded that ZnONPs are a potential anti-proliferative agent against human colon cancer cell lines [152]. Recent research findings on inorganic NPs for colorectal cancer treatment are listed in Table 1.

**Table 1:** Latest investigations on various inorganic NPs for the treatment of colorectal cancer

Type	Therapeutic agent/s	Particle size	Cell lines	Animal model	Route of administration	Ref.
Carbon nanotubes	Amylose derivatives containing poly(L-lysine)	-	Human colon cancer cell line HCT 116	BALB/c nude mice	Intravenous	[153]
Carbon nanotubes	CNT-CpG complex	-	Human colon cancer cell line HCT 116/ Mouse colon cancer cell line CT 26	C57BL/6 mice (38-week-old)	Intraperitoneal	[154]
Carbon nanotubes	5-fluorouracil (5FU), Purpurin (Purp), and 1,8-naphthalimide DNA intercalators (NIDIs)	Outer diameter 44 ± 25 nm/ Inner diameter 12 ± 6 nm	Me 45/ Human colon cancer cell line HCT 116	-	-	[155]
Carbon nanotubes	SN38 Cetuximab	-	HCT116/ HT29/ SW620	-	-	[156]
Quantum dots	Graphene	86 ± 1.35 nm	HCT116	-	-	[157]
Quantum dots	Curcumin	115 nm	HCT 116	-	-	[158]
Quantum dots	Zinc oxide	1–4 nm	HT 29	Wistar rats	Intradermal	[159]
Quantum dots	Unsymmetrical bisacridine derivatives	10–12 nm	HCT 116	-	-	[160]
Mesoporous silica nanoparticles	5-FU	248.59 nm	SW620	-	-	[161]
Mesoporous silica nanoparticles	α-L-fucose targeting lectin Ulex Europaeus Agglutinin-1 (UEA1)	75.4 ± 5 nm	Caco-2/ HCT116	A/J male mice (Six-week-old)	Intraperitoneal	[162]
Mesoporous silica nanoparticles	veratridine	215.5 nm	HCT116/ CCD-33Co	-	-	[163]
Mesoporous silica nanoparticles	Cisplatin		HT 29/ HUV	-	-	[164]
Iron oxide nanoparticles	Curcumin	5–7 nm	HT 29	-	-	[165]
Iron oxide nanoparticles	5-FU	30–100 nm	HT 29	-	-	[166]
Iron oxide nanoparticles	Peptide 1: Boc-Leu-Aib-Val-dPro-lPro-Val-Aib-Leu-OMe and Peptide 2: Boc-U-Gpn-OMe	-	HT 29	-	-	[167]
Gold nanoparticles	Curcumin	45.1 nm	HT 29	-	-	[168]
Gold nanoparticles	Curcumin-Graphene based	15.62 ± 4.04 nm	HT 29/ SW-948	-	-	[169]
Gold nanoparticles	Hibiscus/ curcumin extract	Hibiscus AuNPs: 13 nm/ Curcumin AuNPs: 18.3 nm	HT116	-	-	[170]
Silver nanoparticles	β-sitosterol	4–21 nm	HT 29	-	-	[171]
Silver nanoparticles	*Croton tiglium* L. Seeds Extract	-	-	Adult male Wistar rats	Intraperitoneal	[172]
Zinc oxide nanoparticles	*Swertia chirayita* Leaf Extract	285–209.9 nm	Caco-2/ HCT116	-	-	[173]
Zinc oxide nanoparticles	*Ferula asafetida*	30–50 nm	HT-29	-	-	[174]

## Toxicity aspects of INPs and potential ways to overcome them

The cytotoxic effects of INPs present both advantages and challenges. On one hand, these NPs can effectively kill cancer cells, making them useful in cancer treatment. On the other hand, they can also damage healthy cells by triggering cell apoptosis, necrosis, or autophagy. To maximize the therapeutic potential of INPs, it is essential to design NPs that are selectively toxic to cancer cells while remaining biocompatible and harmless to normal cells. Recently, there has been growing interest in functionalizing INPs with protein molecules and using green synthesis methods (plants, microbes, biodegradable waste, etc.) [175]. These approaches have shown promise in reducing the toxicities associated with INPs, compared to their non-functionalized and chemically synthesized counterparts [21, 176].

An interesting study by Mukherjee and colleagues explored the toxicity differences between green synthesized silver NPs and their chemically synthesized counterparts. They discovered that green synthesized silver NPs exhibited notable anticancer activity across three cancer cell lines: A549, B16F10, and MCF-7. These green synthesized NPs significantly inhibited the proliferation of the cancer cells in a dose-dependent manner, with concentrations ranging from 3 to 30 µM. In contrast, chemically synthesized silver NPs failed to suppress cancer cell proliferation within the same concentration range. Additionally, biocompatibility assessments on human umbilical vein endothelial cells (HUVEC) and Chinese hamster ovary (CHO) cells showed no toxicity for green synthesized NPs within the 3–30 µM range. However, a slight toxicity was observed in rat cardiomyoblast cells (H9C2) at the highest concentration of 30 µM. Conversely, the chemically synthesized NPs exhibited toxicity to these normal cells even at lower doses [177].

Another interesting investigation by Kummara and teammates revealed that chemically synthesized silver NPs were toxic to human dermal fibroblast cells at concentrations between 120 and 240 ppm. Conversely, green synthesized silver NPs demonstrated no toxicity to normal cells within the same concentration range of 0 to 240 ppm. Yet, green synthesized silver NPs effectively inhibited the growth of NCI-H460 lung cancer cells at concentrations of 160, 200, and 240 ppm. In contrast, similar concentrations of chemically synthesized silver NPs did not result in significant inhibition of lung cancer cell proliferation. Additionally, toxicity tests on brine shrimp showed that green synthesized NPs caused 100% mortality at 240 ppm and 56% mortality at 120 ppm. On the other hand, chemically synthesized NPs led to 100% mortality at both 240 ppm and 120 ppm. These results underscore the excellent biocompatibility of green synthesized INPs compared to their chemically synthesized counterparts [178].

Surface functionalization of INPs with proteins has been shown to enhance their biocompatibility through various mechanisms. These include reducing the release of free metal ions, masking residual toxic capping agents, improving water dispersibility, decreasing immunotoxicity, enhancing renal excretion, and lowering thrombogenic activity. Modifying INPs with biocompatible polymers such as proteins enhances their cytocompatibility by mitigating the release of free metal ions, thus reducing ROS generation and related cytotoxic effects [176]. For example, Fe_3_O_4_ NPs coated with silk fibroin exhibited diminished iron ion release and ROS production, facilitating neural cell differentiation instead of apoptosis [179]. Similarly, HSA-coated Gd-DTPA and gelatin-coated CdTe quantum dots showed improved biocompatibility and lower cytotoxicity by minimizing the efflux of toxic ions [180].

An interesting investigation reported that encapsulating CTAB-coated AuNRs with an HSA shell reduced the harmful effects of residual CTAB on cell membranes, resulting in improved biocompatibility with over 91% viability in 4T1 breast cancer cells. Conversely, uncoated AuNRs without albumin exhibited significantly reduced cell viability due to the presence of toxic CTAB residues. A significant obstacle with free CNTs is their propensity to aggregate due to hydrophobic interactions between the sp2 carbon tube shells. Protein coating enhances the water dispersibility of CNTs by breaking these hydrophobic interactions, thus making them safe for mesenchymal stem cells. Likewise, protein functionalization enables the synthesis of photostable QDs, typically created in hydrophobic environments, in aqueous media, thereby facilitating their use in biological systems [181]. In another study, ultra-small BSA-conjugated Gd nanoparticles (less than 3 nm) were identified as valuable MRI contrast agents due to their renal excretion capability. Following intravenous administration, these nanoparticles produced significant negative contrast enhancements in mouse liver MRI images, which returned to baseline after 24 h, primarily due to renal clearance. This excretion process is crucial for reducing potential toxicity in the body [182]. Free and PEGylated CNTs are notably thrombogenic, likely due to their negatively charged surfaces promoting clotting factor activity. Plasma proteins like fibrinogen and von Willebrand factor can bind to CNTs, causing platelet adhesion and activation. Thus, a study showed that modifying the surface of CNTs with HAS inhibits protein adsorption and platelet adhesion, reducing their prothrombotic effects, decreasing the risk of platelet aggregation and thrombus formation, thereby improving their biocompatibility [183].

## Conclusion and future perspectives

CRC stands as a formidable adversary in the realm of oncology. However, amid this challenge, INPs emerge as promising platforms in the fight against this malignancy. They represent a potential paradigm shift in CRC therapy, offering the prospect of addressing and surmounting the limitations entrenched in conventional chemotherapy. INPs surpass organic NPs due to their unique attributes such as heightened photosensitivity, remarkable conductivity, magnetic allure, and thermal proficiency, thus serving dual roles as drug carriers and therapeutic agents. Derived primarily from metals, metal oxides, and non-metallic materials, INPs exhibit superior drug-loading capacity and facilitate advanced photothermal and PDT. As we mentioned earlier in the introduction, to date, only two INP-based products, NanoTherm^®^ and Hensify^®^, have garnered approval by EMA for the treatment of cancer. Additionally, other INP-based formulations like Arimune, Auroshell, etc., are in clinical trials, highlighting the ongoing exploration of INPs in cancer therapy. Yet, no INPs have been approved for colorectal cancer therapy.

Although INP-based approaches to CRC therapy offer substantial benefits, significant hurdles remain before their seamless integration into clinical practice. Understanding the pathways through which INPs are eliminated from the body is crucial for their successful clinical application. While renal elimination via urine is not viable for most INPs due to their non-biodegradable nature and larger particle sizes, hepatobiliary elimination through feces emerges as a potential alternative route. However, this elimination process is influenced by INPs’ interactions with liver cells, impacting their fate. The complex journey of INPs through liver sinusoids, hepatocytes, bile ducts, intestines, and eventual excretion via feces underscores the intricate dynamics involved. Extensive investigation into biosafety, biodegradation, biodistribution, excretion, and in vivo behavior of inorganic nanomaterials is imperative due to the limited data available regarding their safety profiles. While concerns remain over the toxicity associated with inorganic NPs, particularly at higher concentrations, strategic measures such as utilizing biologically sourced materials in NP synthesis and encasing them within degradable polymers/lipids hold promise in mitigating these risks. Moreover, surface functionalization of INPs with biological materials introduces further safety considerations, amplifying their potential interactions with tissues and biomolecules upon in vivo administration. Nevertheless, ensuring homogeneity in INPs is crucial; yet, scaling up production while preserving reproducibility and uniformity presents a significant challenge, especially for drug-loaded complex targeted INPs. Despite both advantages and limitations, ample opportunities exist for further research on INPs for efficient CRC therapy, while also considering toxicity and clinical translation. Consequently, this review is poised to offer significant assistance to scientists and researchers engaged in CRC research.

## Data Availability

No datasets were generated or analysed during the current study.
